# Direct and quantitative analysis of altered metabolic flux distributions and cellular ATP production pathway in fumarate hydratase-diminished cells

**DOI:** 10.1038/s41598-020-70000-6

**Published:** 2020-08-03

**Authors:** Shingo Noguchi, Hirokazu Ishikawa, Kenichi Wakita, Fumio Matsuda, Hiroshi Shimizu

**Affiliations:** 1 Biomarker Department, Daiichi Sankyo Co., Ltd, Tokyo, Japan; 2 Department of Bioinformatic Engineering, Graduate School of Information Science and Technology, Osaka University, Osaka, Japan; 3 Biological Research Department, Daiichi Sankyo RD Novare Co., Ltd, Tokyo, Japan

**Keywords:** Computational biology and bioinformatics, Biochemistry, Biotechnology, Metabolomics, Cancer, Cancer metabolism

## Abstract

Fumarate hydratase (FH) is an enzyme in the tricarboxylic acid (TCA) cycle, biallelic loss-of-function mutations of which are associated with hereditary leiomyomatosis and renal cell cancer. However, how FH defect modulates intracellular metabolic fluxes in human cells has remained unclear. This study aimed to reveal metabolic flux alterations induced by reduced FH activity. We applied ^13^C metabolic flux analysis (^13^C-MFA) to an established cell line with diminished FH activity (FH^dim^) and parental HEK293 cells. FH^dim^ cells showed reduced pyruvate import flux into mitochondria and subsequent TCA cycle fluxes. Interestingly, the diminished FH activity decreased FH flux only by about 20%, suggesting a very low need for FH to maintain the oxidative TCA cycle. Cellular ATP production from the TCA cycle was dominantly suppressed compared with that from glycolysis in FH^dim^ cells. Consistently, FH^dim^ cells exhibited higher glucose dependence for ATP production and higher resistance to an ATP synthase inhibitor. In summary, using FH^dim^ cells we demonstrated that FH defect led to suppressed pyruvate import into mitochondria, followed by downregulated TCA cycle activity and altered ATP production pathway balance from the TCA cycle to glycolysis. We confirmed that ^13^C-MFA can provide direct and quantitative information on metabolic alterations induced by FH defect.

## Introduction

Fumarate hydratase (FH) is one of the enzymes in the tricarboxylic acid (TCA) cycle and catalyses the hydration of fumarate to malate. FH gene mutations causing loss of function are known to be associated with predispositions to hereditary leiomyomatosis and renal cell cancer (HLRCC)^1,2^. This syndrome is characterised by cutaneous and uterine leiomyoma and an aggressive form of type 2 papillary renal carcinoma, which is often fatal^3^. Since the TCA cycle plays an important role in cellular energy metabolism, FH defect leads to significant metabolic reprogramming. Effects of FH defect on cellular metabolism have been well studied using Fh1 (murine FH)-knockout mouse cells and FH-deficient UOK262 cells derived from HLRCC-associated kidney tumour^4^. Yang et al., Frezza et al. and O’Flaherty et al. indirectly demonstrated the downregulated mitochondrial metabolism and the upregulated glycolysis in FH-inactivated cells by measuring the oxygen consumption rate (OCR) and extracellular acidification rate (ECAR)^5–7^. Via metabolome analysis, Adam et al. and Zheng et al. revealed that FH inactivation altered urea cycle metabolism and caused arginine auxotrophy^8,9^. In addition, Gonçalves et al. demonstrated the increased phosphorylation of pyruvate dehydrogenase and restriction of carbon entry from glucose to the TCA cycle in FH-deficient cells by phosphoproteome analysis and ^13^C tracer analysis^10^. As described above, how FH defect modulates cellular energy metabolism has been addressed by various approaches. However, to the best of our knowledge, a ^13^C metabolic flux analysis (^13^C-MFA)-based approach has not been applied to address the impact of FH defect on cellular metabolism.

^13^C-MFA is a powerful tool for quantifying intracellular metabolic flux. It computes the intracellular metabolic flux distribution using a mathematical model by integrating nutrient uptake and secretion rates with the ^13^C labelling pattern of intracellular metabolites^11^. Moreover, estimates of cofactor information on energy metabolism, such as NADH, NADPH or ATP production/consumption flux, can be obtained by this analysis. Therefore, ^13^C-MFA is expected to provide direct and quantitative information on altered cellular metabolism induced by FH defect.

In the present study, we revealed how FH defect affects cellular metabolism by comparing metabolic flux distributions within a pair of isogenic cell lines: HEK293 cells with wild-type FH activity and their counterparts with diminished FH activity.

## Results

### Characterisation of the generated cell line with diminished FH activity

**Figure 1:**
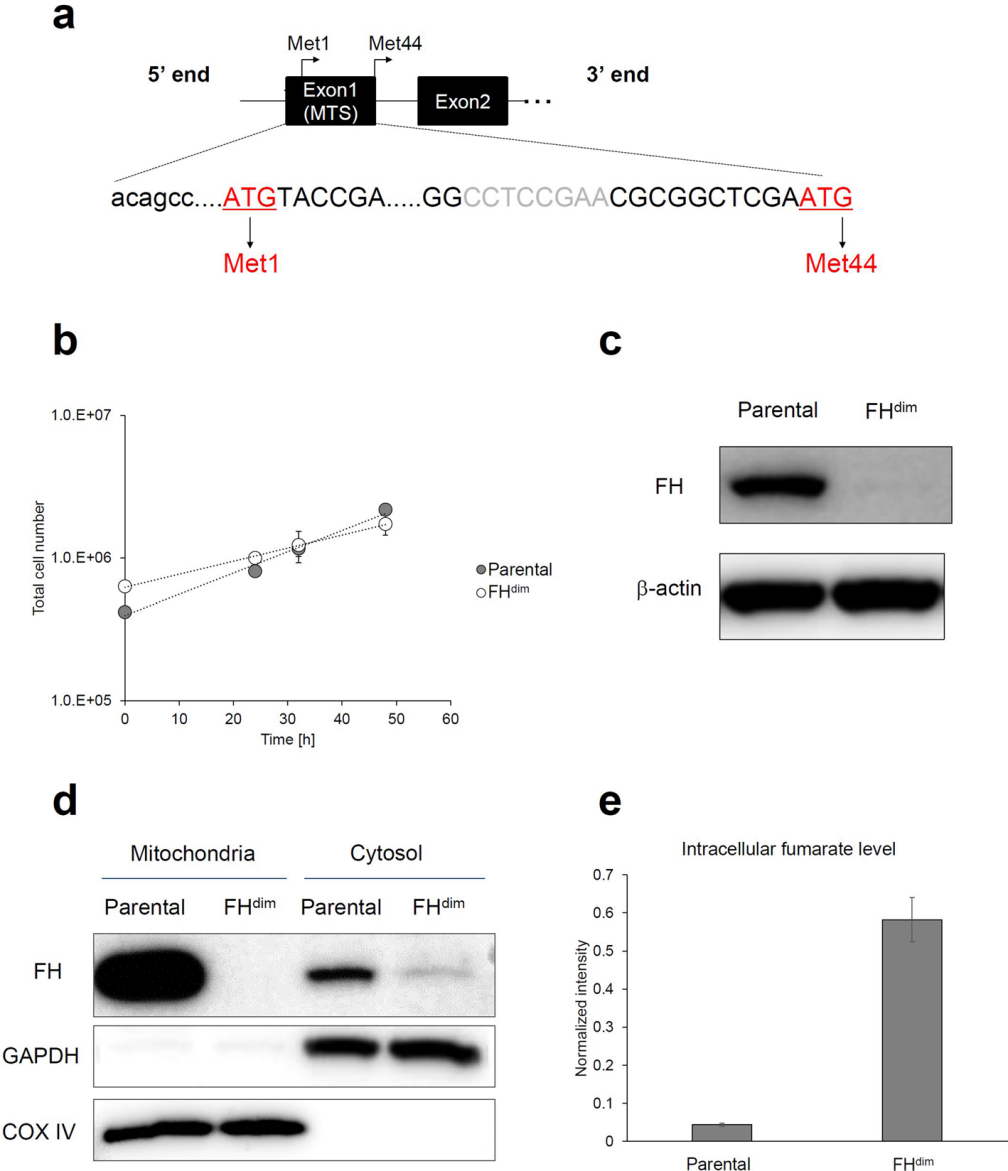
Characterization of FH^dim^ cells. (**a**) Schematic representations of the FH gene. Exon 1 contains mitochondrial targeting sequence (MTS). Capital letters and grey letters represent FH coding sequence and deleted sequence in FH^dim^ cells, respectively. (**b**) Growth curves of parental and FH^dim^ cells. (**c**) FH protein expression of parental and FH^dim^ cells. β-Actin was used as an internal control. (**d**) Mitochondrial and cytosolic FH expression. Glyceraldehyde-3-phosphate dehydrogenase (GAPDH) and cytochrome C oxidase subunit 4 (COX IV) were used as cytosolic and mitochondrial markers, respectively. In (**c**) and (**d**), cropped images were displayed. Full uncropped blots are presented in Supplemental Figure S3. (**e**) Intracellular fumarate levels in parental and FH^dim^ cells. Each data point, bar chart and error bar represent the mean and standard deviation from triplicate samples.

To investigate the effects of diminished FH activity on central carbon energy metabolism, we generated HEK293 cells with knockout of FH exon 1 via an 8-bp frameshift deletion (FH^dim^) using the CRISPR-Cas9 system (Fig. 1a).

FH^dim^ cells showed a decrease in the specific cell growth rate (Fig. 1b; Parental: 0.0343 h^−1^, FH^dim^: 0.0208 h^−1^). Since both cells showed exponential growth until 48 h, they were considered to be in a metabolically steady state necessary for ^13^C-MFA^12^. Western blot analysis of whole-cell lysate confirmed that FH protein expression in FH^dim^ cells was rarely detected compared with that in parental cells (Fig. 1c). FH proteins are distributed in both mitochondria and cytosol, which are referred to echoforms^13^. We separated mitochondria and cytosol to evaluate the FH protein expression and enzyme activity in each compartment. Neither protein expression nor enzyme activity of FH was detected in mitochondria (Fig. 1d and Table 1). Unexpectedly, FH protein expression was clearly detected in the cytosol, and FH enzyme activity was maintained at about 44% of the level of parental cells (Fig. 1d and Table 1). FH exon 1 contains a mitochondrial targeting sequence (MTS) and two in-frame ATGs encoding Met1 and Met44 (Fig. 1a). The cytosolic FH echoform is expressed from the secondary in-frame ATG encoding Met44^14^. Since the 8-bp deletion was located between the two ATGs, FH^dim^ cells still had the secondary in-frame ATG, making it possible to produce the FH cytosolic echoform (Fig. 1a). To confirm the diminishment of the intracellular FH activity in FH^dim^ cells with remaining FH cytosolic echoform, we measured the intracellular fumarate level by gas chromatography mass spectrometry (GC–MS). We found that the fumarate level increased about 13-fold compared with that of parental cells, which suggested the reduction of intracellular FH activity in FH^dim^ cells (Fig. 1e).

**Table 1:** Mitochondrial and cytosolic FH enzyme activity.

Compartment	Cell	FH activity (nmol/min/mg-protein)
Mitochondria	Parental	14.1 ± 0.6
	FH^dim^	ND
Cytosol	Parental	2.7 ± 0.1
	FH^dim^	1.2 ± 0.5

FH activity is represented as the mean ± standard deviation from triplicate samples. ND: not detected.

### Extracellular flux profiling

The levels of uptake or secretion fluxes of glucose, organic acids and amino acids were calculated based on the specific growth rates and time course profiles of extracellular metabolite concentrations. In FH^dim^ cells, glucose uptake flux decreased by 8% (Table 2). The secretion fluxes of lactate and pyruvate also decreased by 7% and 6%, respectively (Table 2). The ratio of lactate secretion flux to glucose uptake flux was comparable between the two cells (Parental: 1.88, FH^dim^: 1.89; Table 2), which indicates that the diminished FH activity had little effect on glycolysis. As with most mammalian cells, HEK293 cells require glutamine for growth^15^. Glutamine is an important carbon or nitrogen source for the production of other amino acids and for the TCA cycle. Glutamine uptake flux was markedly decreased by 36% in FH^dim^ cells (Table 2), suggesting the occurrence of alterations of amino acid metabolism or TCA cycle metabolism. Moreover, proline (Pro) secretion flux was dramatically decreased by 89% in FH^dim^ cells (Table 2), suggesting the downregulation of Pro synthesis flux.

**Table 2:** Estimated extracellular flux.

Metabolite	Parental		FH^dim^		Percent change (%)
	Flux (nmol/10^6^ cells/h)	95% CI (LB, UB)	Flux (nmol/10^6^ cells/h)	95% CI (LB, UB)	
**Uptake**					
Glucose	578.8	(541.5, 616.1)	533.5	(499.3, 567.8)	− 8
Glutamine	82.7	(77.3, 88.1)	52.7	(49.3, 56.1)	− 36
Cysteine	4.1	(3.8, 4.5)	3.3	(3.1, 3.6)	− 19
Serine	22.0	(20, 24.1)	16.2	(15.2, 17.3)	− 26
Arginine	8.7	(8.1, 9.3)	9.6	(8.9, 10.4)	10
**Secretion**					
Lactate	1,087.7	(1,036.9, 1,138.5)	1,009.8	(975.1, 1,044.6)	− 7
Pyruvate	61.7	(55.5, 67.8)	57.8	(52, 63.6)	− 6
Alanine	17.1	(16.2, 18.1)	19.4	(18.4, 20.3)	13
Proline	13.9	(13.4, 14.3)	1.5	(1.4, 1.5)	− 89
**Ratio of lactate secretion flux to glucose uptake flux**	1.88		1.89		

Percent change is defined as the ratio of the difference between parental and FH^dim^ cells to the flux value in parental cells. Positive or negative value means increased or decreased flux in FH^dim^ cells, respectively. CI, confidence interval; LB, lower bound; UB, upper bound.

### Mass isotopomer distributions of parental and FH^dim^ cells fed with [1,2-^13^C]glucose and [U-^13^C]glutamine

**Figure 2:**
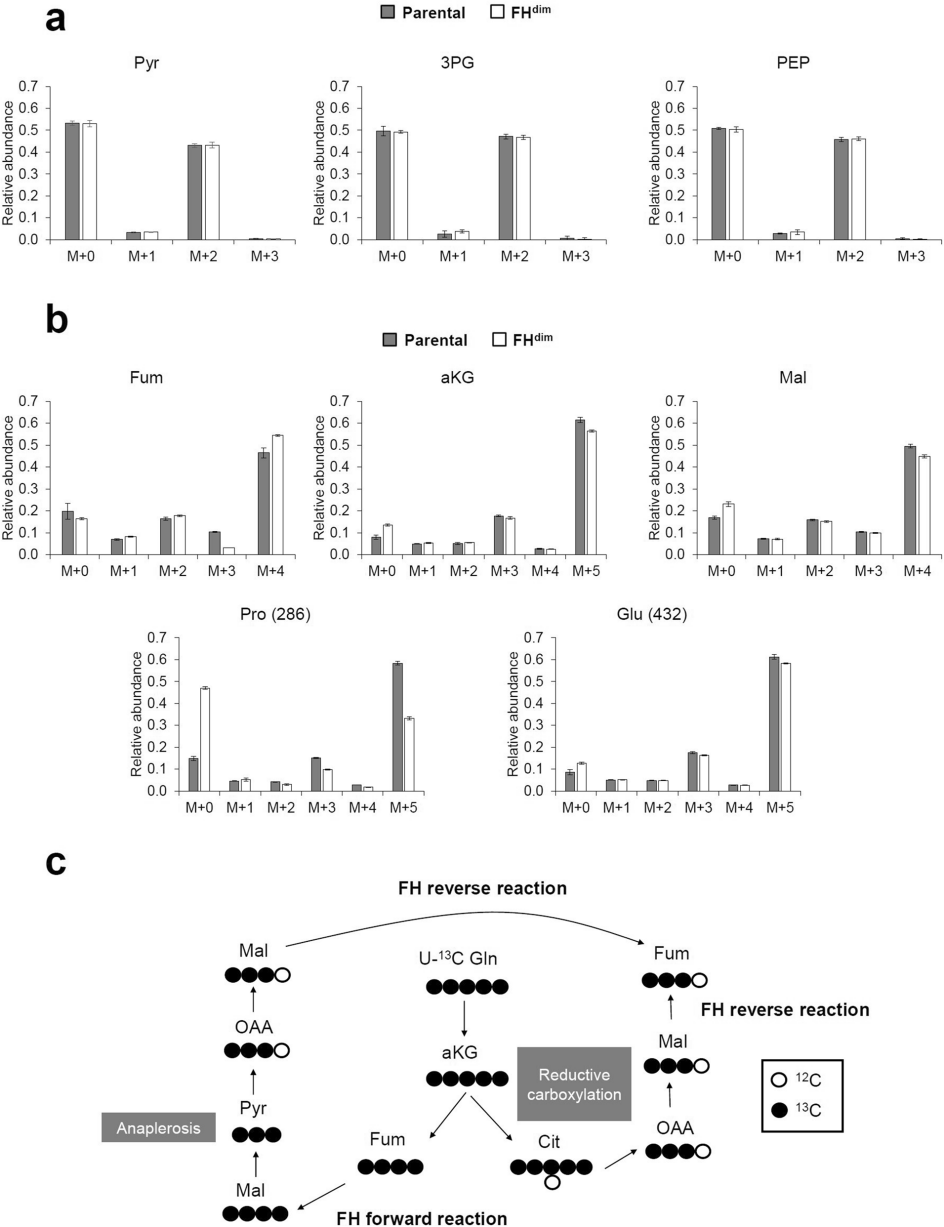
^13^C labelling patterns of intracellular metabolites. Mass isotopomer distribution in parental and FH^dim^ cells fed with [1,2-^13^C]glucose (**a**) and [U-^13^C]glutamine (**b**). Each bar chart and error bar represent the mean and standard deviation from triplicate samples. Panel (**c**) illustrates how M + 3 fumarate is generated by U-^13^C glutamine labelling. Abbreviations: Pyr, pyruvate; 3PG, 3-phosphoglyceric acid; PEP, phosphoenolpyruvate; Fum, fumarate; aKG, alpha-ketoglutarate; Mal, malate; Pro, proline; Glu, glutamate; Gln, glutamine; Cit, citrate; OAA, oxaloacetate.

To investigate intracellular metabolism, we measured the change of ^13^C labelling pattern of intracellular metabolites in parental and FH^dim^ cells cultured in medium supplemented with [1,2-^13^C]glucose or [U-^13^C]glutamine. Both cells were metabolically quenched at 24, 29 and 32 h after the addition of ^13^C-labelled carbon sources, and extracted intracellular metabolites were derivatised and subsequently analysed by GC–MS. Measured mass isotopomer distributions (MIDs) were corrected for natural isotope abundance. The time course profiles of MIDs in each metabolite indicated that both cells reached isotopically steady states (Supplemental Figure S1 and Figure S2). The oxidative pentose phosphate pathway (oxPPP) produces M + 1 glycolytic intermediates from [1,2-^13^C]glucose. Both cells showed the production of M + 1 phosphoenolpyruvate (PEP) and 3-phosphoglyceric acid (3PG) under culture with [1,2-^13^C]glucose (Fig. 2a). These results suggest that oxPPP is active in both cells. The M + 1 isotopomer ratios of PEP and 3PG were comparable between parental and FH^dim^ cells, suggesting that the diminished FH activity did not affect oxidative branch flux. Moreover, the MIDs of the glycolytic intermediates (PEP, 3PG and pyruvate) of parental and FH^dim^ cells were very similar to each other (Fig. 2a), indicating that no additional carbon incorporation other than that of glucose occurred in FH^dim^ cells. Regarding malate, the M + 4 isotopomer was dominant in both cells under [U-^13^C]glutamine labelling conditions (Fig. 2b). Since M + 4 malate is mainly produced through the FH forward reaction, FH^dim^ cells are considered to exert intracellular FH enzyme activity. The M + 3 isotopomer ratio of fumarate decreased in FH^dim^ cells (Fig. 2b). M + 3 fumarate is produced from M + 3 malate through an FH reverse reaction via (1) anaplerotic reactions as malic enzyme and pyruvate carboxylase or (2) reductive carboxylation of glutamine (Fig. 2c). Thus, the decrease in M + 3 fumarate suggests decreased flux of FH reverse reaction in FH^dim^ cells, which may be due to a strong driving force for FH forward reaction induced by the increased intracellular fumarate level.

In FH^dim^ cells under culture with [U-^13^C]glutamine, M + 0 Pro increased while M + 5 Pro decreased (Fig. 2b). Glutamate (Glu) is one of the precursors for Pro synthesis, so the decreased M + 5 Pro indicates the downregulation of Pro synthesis flux from Glu.

### Metabolic flux alterations through diminished FH activity

We determined the intracellular flux distribution by fitting the observed MID data to the simulated MID results based on a developed metabolic model. A significant difference in flux between parental and FH^dim^ cells was defined as no overlap in the 95% confidence intervals.

**Figure 3:**
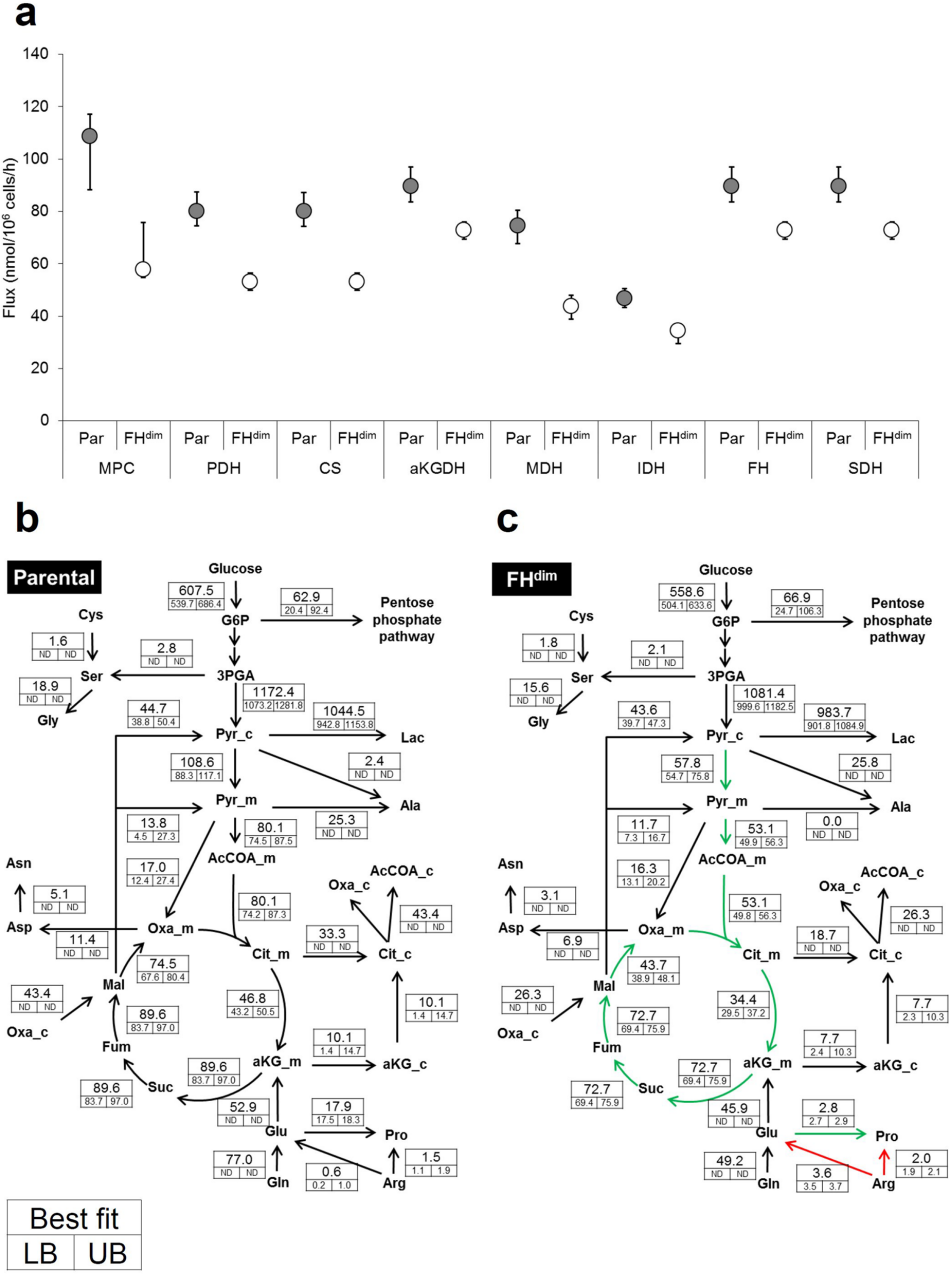
Metabolic flux distributions in parental and FH^dim^ cells. (**a**) Determined metabolic flux of TCA cycle-related metabolic reactions. Error bar represents the 95% confidence interval. Determined metabolic flux distributions in parental (**b**) and FH^dim^ cells (**c**). Flux values are represented as best fitted and 95% confidence intervals (LB, lower boundary; UB, upper boundary; ND, not determined). The unit of flux values is nmol/10^6^ cells/h. Green and red arrows represent significantly decreased and increased fluxes. Significance was defined by no overlap of 95% confidence intervals between parental and FH^dim^ cells. Abbreviations: MPC, mitochondrial pyruvate carrier; PDH, pyruvate dehydrogenase; CS, citrate synthase; aKGDH, alpha-ketoglutarate dehydrogenase; MDH, malate dehydrogenase; IDH, isocitrate dehydrogenase; FH, fumarase; SDH, succinate dehydrogenase.

FH^dim^ cells showed significantly decreased metabolic fluxes in TCA cycle reactions such as those involving pyruvate import into mitochondria (MPC), pyruvate dehydrogenase (PDH), citrate synthase (CS), isocitrate dehydrogenase (IDH), alpha-ketoglutarate dehydrogenase (aKGDH), succinate dehydrogenase (SDH), FH and malate dehydrogenase (MDH) (Fig. 3a). Regarding glutaminolysis, glutamine uptake flux and Glu synthesis flux were decreased by 36% in FH^dim^ cells (Table 2, Fig. 3b, c). However, aKG synthesis flux from Glu was comparable between the two cell types (52.9 nmol/10^6^ cells/h in parental cells and 45.9 nmol/10^6^ cells/h in FH^dim^ cells) (Fig. 3b, c). Interestingly, Pro synthesis flux from Glu was dramatically decreased in FH^dim^ cells (2.8 nmol/10^6^ cells/h) compared with that in parental cells (17.9 nmol/10^6^ cells/h) (Fig. 3b, c). Moreover, anaplerotic pyruvate carboxylase flux remained unchanged (Fig. 3b, c). These results imply that the diminished FH activity leads to impaired TCA cycle activity through the suppression of pyruvate import into mitochondria. The ratio of FH reverse reaction flux to FH forward reaction flux decreased in FH^dim^ cells (Parental: 1.00, FH^dim^: 0.18; Supplemental Table S1), which means that the FH forward reaction is dominant in FH^dim^ cells. This result is in line with the MID data for fumarate, as mentioned in the previous section.

Intracellular NAD^+^ is reduced to NADH by the TCA cycle. This NADH is utilised for oxidative phosphorylation (OxPHOS)-dependent ATP production. Thus, the impaired TCA cycle activity may affect cellular ATP production. We calculated net ATP production flux in both types of cell based on OxPHOS flux and metabolic fluxes responsible for ATP production and consumption. Net ATP production flux decreased in FH^dim^ cells by 330.2 nmol/10^6^ cells/h (Table 3), which is consistent with the decreased growth rate of FH^dim^ cells (Fig. 1b). Next, we estimated the contribution of the glycolytic pathway and TCA cycle to cellular ATP production. Glycolysis- and TCA cycle-derived ATP production fluxes were decreased in FH^dim^ cells by 86.7 nmol/10^6^ cells/h and 244.1 nmol/10^6^ cells/h, respectively (Table 3). Thus, the decrease in net ATP production flux in FH^dim^ cells is mainly caused by the decrease in ATP production flux from the TCA cycle.

**Table 3:** Calculated ATP production flux based on results of ^13^C metabolic flux analysis.

Pathway	ATP production flux (nmol/10^6^ cells/h)			Fold change
	Parental	FH^dim^	Difference	FH^dim^/Parental
Glycolysis	1,161.3	1,074.6	86.7	0.93
TCA cycle	1,070.9	826.8	244.1	0.77
Total net	2,215.3	1,885.1	330.2	0.85

### Glycolytic shift in ATP production by diminished FH activity

**Figure 4:**
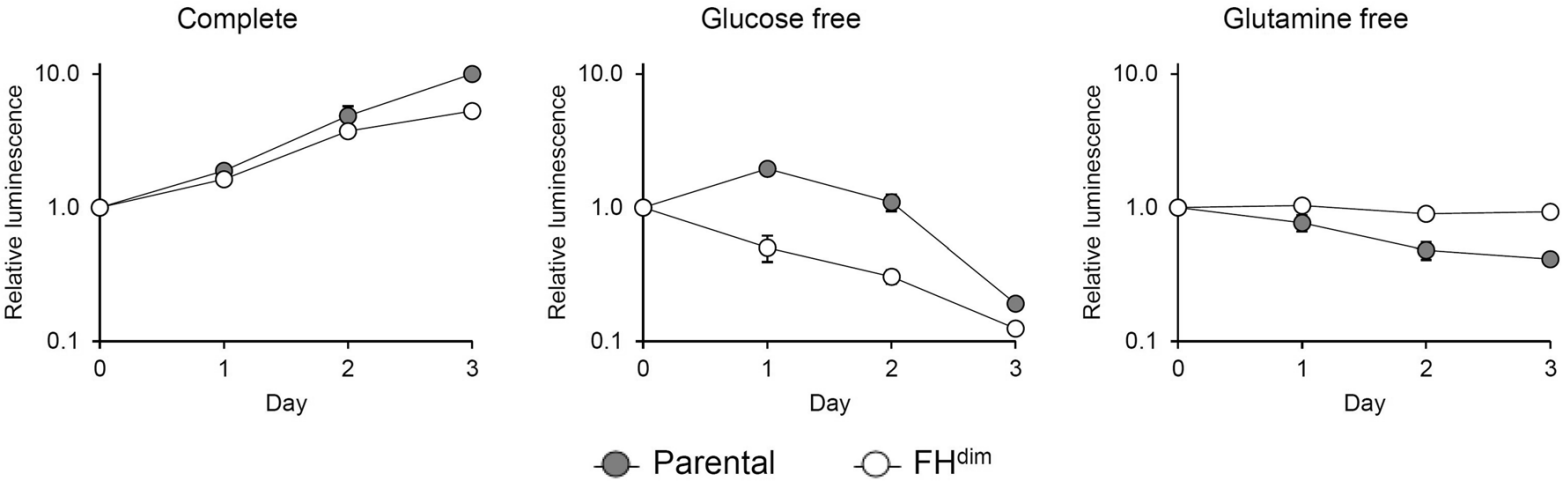
Time course profile of cellular ATP levels of parental and FH^dim^ cells in glucose- or glutamine-free conditions. Each data point and error bar represent the mean and standard deviation from quadruplicate samples.

**Figure 5:**
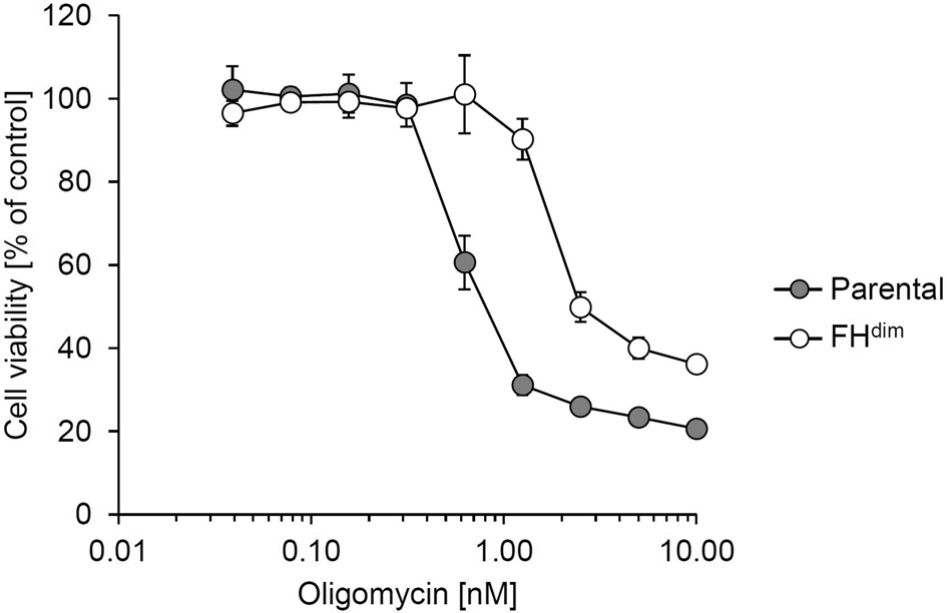
Inhibitory activity of oligomycin on the viability of parental and FH^dim^ cells. Each data point and error bar represent the mean and standard deviation from triplicate samples.

As described above, we observed the downregulation of TCA cycle activity and the reduction of TCA cycle-derived ATP production flux in FH^dim^ cells. Based on these findings, we hypothesised that glycolysis is more dominant than the TCA cycle for cellular ATP production in FH^dim^ cells. To confirm this, we cultured both cells under glucose-free or glutamine-free conditions and investigated cellular ATP production. Cells cultured without glucose supplementation are forced to rely on processes other than glycolysis for the production of cellular ATP. In the case without glutamine supplementation, TCA cycle-derived cellular ATP production is suppressed since glutamine is the predominant fuel for the TCA cycle. Under complete medium conditions, parental and FH^dim^ cells showed continuously increasing cellular ATP levels (Fig. 4). However, under glucose-free conditions, parental cells showed an increasing cellular ATP level on day 1, followed by a decrease in the ATP level until day 3, while the ATP level continuously decreased in FH^dim^ cells from day 1 to day 3 (Fig. 4). In contrast, under glutamine-free conditions, parental cells showed a decreasing cellular ATP level until day 3, while FH^dim^ cells maintained the cellular ATP level until day 3 (Fig. 4). Moreover, FH^dim^ cells were more resistant to oligomycin, an inhibitor of mitochondrial ATP synthase, than parental cells (Fig. 5). These results suggest that the diminished FH activity renders the cellular ATP production more dependent on glycolysis and less dependent on the TCA cycle.

## Discussion

In this study, we developed isogenic cell lines with diminished FH activity from HEK293 cells using the CRISPR-Cas9 system (Fig. 1) and investigated the impact of this diminished activity on central carbon metabolism by ^13^C-MFA using [1,2-^13^C]glucose and [U-^13^C]glutamine (Fig. 3). An isogenic cell line pair is a good tool for analysing the effects of a specific gene alteration since the paired cell lines share an identical genetic background. The developed FH^dim^ cells showed the retention of FH enzyme expression and activity only in the cytosolic fraction (Fig. 1). Importantly, mitochondrial and FH cytosolic echoforms are encoded by a single FH gene^13^. Since the FH gene promoter has various transcription start sites, two types of FH mRNA are transcribed: one is translated into FH protein containing MTS, while the other is translated into that lacking MTS^14^. These two types of transcript lead to the dual localisation of FH protein. FH^dim^ cells are unable to express the FH mitochondrial echoform due to the frameshift deletion within the MTS coding region, while the FH cytosolic echoform can be expressed due to the secondary ATG, from which translation of the FH cytosolic echoform is initiated. Indeed, Dik et al. transfected FH cDNA with a frameshift within the MTS coding region to HEK293T cells, and they detected exogenous FH protein expression restricted to the cytosol^14^. This supports the phenomenon observed in our FH^dim^ cells.

Previously, it was reported that FH inactivation caused the upregulation of glycolysis^4–7^. However, our FH^dim^ cells developed in this study did not show such a phenotype (Table 2, Fig. 3). A suggested mechanism for the glycolysis upregulation induced by FH inactivation is through the stabilisation of hypoxia-inducible factor (HIF). This stabilisation is regulated by the inhibition of HIF prolyl hydroxylase via accumulated intracellular fumarate^16,17^. The increase of intracellular fumarate level in our FH^dim^ cells was about 13-fold relative to that of parental cells, which is much lower than that of Fh1-knockout mouse cells (about 100-fold) as described in a previous report^6^. We assume that the degree of increase is insufficient to stabilise HIF. Moreover, O’Flaherty et al. reported that cytosolic Fh1 protein expression was sufficient to reduce intracellular fumarate level and to restore the upregulated HIF pathway^7^. Thus, the cytosolic FH expression remaining in FH^dim^ cells (Fig. 1) may suppress the HIF-dependent upregulation of glycolysis. Interestingly, the cytosolic FH enzyme activity remaining in FH^dim^ cells (Fig. 1) was sufficient to drive the TCA cycle without truncation despite drastically reduced total FH enzyme activity (Fig. 1, Table 1 and Fig. 3). This implies that the FH protein expression level is high in our model, which is an important finding because FH is known to be a tumour suppressor, and fumarate has been reported as an oncometabolite^18,19^. The required FH expression level for maintaining the oxidative TCA cycle is much lower than the actual expression level, which preserves the anti-tumour function of FH until complete loss of function. Hence, the findings from this model do not explain tumorigenesis by FH loss of function, but provide very important evidence to understand the function of FH.

We demonstrated alterations in intracellular metabolic flux distributions induced by the diminished FH activity, including downregulation of TCA cycle flux (Fig. 3). The observed TCA cycle downregulation mainly resulted from suppressed pyruvate import flux into mitochondria (Fig. 3). Gonçalves et al. reported that pyruvate dehydrogenase activity was inhibited through its phosphorylation in UOK262 cells^10^, which is consistent with the results observed in this study (Fig. 3). Besides TCA cycle flux, Pro synthesis flux was also decreased in FH^dim^ cells (Fig. 3), which is in line with the previous observation that Pro secretion flux decreased in Fh1 knockout mouse cells^20^. To the best of our knowledge, no reports showing that diminished FH activity suppresses Pro synthesis flux derived from glutamine have been published. Intracellular Pro is synthesised from Glu through the formation of 1-pyrroline-5-carboxylate (P5C) as an intermediate^21^, and mitochondrial P5C reductase 1 (PYCR1) uses NADH as a cofactor for Pro synthesis with a higher preference compared with NADPH^22^. Given the decreased NADH production flux in FH^dim^ cells induced by downregulation of the TCA cycle, the reduction of Pro synthesis flux is considered to be due to redox imbalance. Indeed, Pro synthesis has been reported to be associated with the maintenance of redox balance^23,24^. Net NADPH consumption flux in FH^dim^ cells was lower than that in parental cells (Supplemental Table S4). This is because flux of lipid synthesis and proline synthesis from glutamate decreased in FH^dim^ cells. Since FH inactivation increases ROS generation through NADPH oxidase^25^^,^ increased net NADPH production might be a result of compensating the enhanced NADPH oxidase. Our finding warrants further investigation using other model cells and clinical samples to study FH-loss cancer dependence on proline metabolism and explore metabolic vulnerabilities leading to new therapeutic interventions.

FH-deficient cells are known to be resistant to respiratory chain inhibitors such as oligomycin since accumulated intracellular fumarate inhibits respiratory chain complexes I and II^26^. In this study, we revealed that TCA cycle-derived ATP production was decreased in FH^dim^ cells based on cofactor balance calculated from the ^13^C-MFA results (Table 3). Moreover, we confirmed that the ATP level in FH^dim^ cells was decreased more than that in parental cells under glucose-free conditions (Fig. 4) and that FH^dim^ cells were more tolerant of oligomycin treatment (Fig. 5). Thus, the diminishment of FH activity leads to a glycolytic shift in cellular ATP production due to the TCA cycle defect and to the increased resistance to the respiratory chain inhibitor. These results shed more light on the mechanism of resistance to respiratory chain inhibitors as described in previous study^26^. Our results also raise the possibility of FH functioning in the tolerance of hypoxia, which is an important mechanism in tumorigenesis. Further studies on metabolic flux alterations considering tumor microenvironment as gradients of nutrition and oxygen^27,28^ could provide informative insights into a role of FH under tumor hypoxia. Although previous findings indirectly suggested the upregulation of glycolysis and downregulation of OxPHOS in FH-inactivated cells by measuring ECAR and OCR^5–7^, this study directly indicated the alterations of the dominant cellular ATP production source induced by the diminishment of FH activity. Furthermore, ^13^C-MFA results showed that the ratio of FH reverse reaction flux to FH forward reaction flux decreased in FH^dim^ cells (Supplemental Table S1). This indicates that the intracellular accumulation of fumarate induced by diminished FH activity drives the FH forward reaction in FH^dim^ cells. As described above, ^13^C-MFA can provide informative results that promote understanding of cellular energy metabolism other than intracellular metabolic flux distributions.

In summary, we developed FH^dim^ cells with diminished FH activity and clarified its role by applying ^13^C-MFA. We demonstrated how FH defect altered metabolic flux distributions and the cellular ATP production pathway directly and quantitatively. Our results should provide helpful insights for future research to understand the pathology of HLRCC and develop new therapeutic strategies.

## Methods

### Generation of the cell line with diminished FH activity using CRISPR-Cas9 system

HEK293 cells (purchased from American Type Culture Collection) were cultured in Dulbecco’s modified Eagle’s medium (DMEM) supplemented with 10% foetal bovine serum (FBS). HEK293 cells were transfected with Edit-R Cas9 Expression Plasmid (GE Healthcare, Inc.) and sgRNA expression plasmid using Lipofectamine2000. Subsequently, monoclonal cells were obtained by limiting dilution. Genomic DNA from the monoclonal cells including FH exon 1 was amplified with the following selection primers: forward, 5′-TTGGATAAGAGCGGAGGCCGGTGGG-3′, reverse, 5′-CGGGAGCGGGCCCAGTAGGACCCTC-3′. The cells with successful editing of the FH gene were selected by direct DNA sequencing analysis from the amplified fragments.

### Cell culture and ^13^C-labelling experiment

Parental HEK293 cells and FH^dim^ cells were cultured in DMEM without glucose, glutamine, phenol red, sodium pyruvate and sodium bicarbonate (Sigma-Aldrich Co., LLC.) supplemented with 20 mM glucose, 2 mM glutamine, 44 mM sodium bicarbonate and 10% dialysed FBS (Thermo Fisher Scientific, Inc.), unless otherwise mentioned. Parental and FH^dim^ cells were seeded into a 60 mm dish at 5.0 × 10^5^ cells/dish and 8.0 × 10^5^ cells/dish, respectively, followed by culture at 37 °C and 5% CO_2_ in air. The culture medium was replaced with fresh medium at 16 h after cell seeding (this time point was set as 0 h). Cell counting was performed using a TC20 Automated Cell Counter (Bio-Rad Laboratories, Inc.). Each specific growth rate of both types of cell was determined based on a semi-logarithmic plot of total cell number versus time. For the ^13^C-labelling experiments, the culture medium was replaced with one containing [1,2-^13^C]glucose (99% purity; Cambridge Isotope Laboratories, Inc.) or [U-^13^C]glutamine (98% purity; Sigma-Aldrich Co., LLC).

### Extracellular flux determination

One millilitre of the culture supernatant was collected by centrifugation at 10,000×*g* and 4 °C for 5 min. The sample was mixed with an internal standard mixture containing 50 mM pimelate and 5 mM norvaline at a ratio of 9:1. The sample components were separated on an Aminex HPX-87H column (Bio-Rad Laboratories, Inc.). The mobile phase was 1.5 mM H_2_SO_4_ solution. The flow rate and column temperature were 0.5 mL/min and 65 °C, respectively. Amino acids in the medium were measured using a UPLC system (Waters Corp.) by the AccQ.Tag method^29^. Extracellular uptake and excretion rates were determined by nonlinear regression using R version 3.4.1 based on the following equation^30^:

$$A = A_{0} e^{ - kt} + \frac{{q\left( {e^{ - kt} + e^{\mu t} } \right)X_{0} }}{\mu + k}$$

where A and A_0_ are the amount of the corresponding component and its initial amount, k is the first-order degradation rate, µ is the specific growth rate, X_0_ is the initial cell number and q is the specific uptake rate. Since glutamine is spontaneously degraded into pyroglutamate and ammonium in the culture medium^31,32^, we incorporated the first-order degradation rate to calculate uptake rate of glutamine. The degradation rate of glutamine was determined to be 0.00345 h^−1^ by measuring the disappearance of glutamine in the absence of cells. Determined extracellular fluxes of glucose, lactate, pyruvate and amino acids were listed in Table 2.

### Metabolite extraction and GC–MS analysis

Cultured cells were washed with Dulbecco’s phosphate-buffered saline (DPBS) and 0.4 mL of cold methanol (− 80 °C) containing an internal standard (10 μM ribitol) was added to the culture dish. The cells were scraped and collected in a microtube. Furthermore, 0.4 mL of the cold methanol was added to the dish and the remaining cells were retrieved in the microtube. After vortexing vigorously, the tube was centrifuged at 10,000×*g* and 4 °C for 5 min, after which the resulting supernatant was collected. This supernatant was then mixed with 0.3 mL of chloroform and 0.3 mL of water, followed by vigorous vortexing and centrifugation at 12,000×*g* and 4 °C for 5 min. The resulting aqueous layer was collected and dried with an evaporator. To measure the intracellular fumarate level, the dried sample was dissolved in 25 μL of 20 mg/mL methoxyamine hydrochloride (Sigma-Aldrich Co., LLC) in pyridine and incubated for 90 min at 30 °C. Next, 25 μL of *N*-methyl-*N*-trimethylsilyltrifluoroacetamide (MSTFA; GL Sciences, Inc.) was added and incubated for 30 min at 37 °C. To analyse the ^13^C-labelled intracellular metabolites, 25 μL of 20 mg/mL methoxyamine hydrochloride in pyridine was added to the sample and incubated for 60 min at 30 °C. Next, 25 μL of *N*-methyl-*N*-(*tert*-butyldimethylsilyl) trifluoroacetamide (MTBSTFA) + 1% *tert*-butyldimethylchlorosilane (Thermo Fisher Scientific, Inc.) was added and incubated for 30 min at 60 °C. The derivatised sample was analysed using GC–MS (Shimadzu Corp.) equipped with a DB-5MS + DG capillary column (30 m × 0.25 mm i.d. × 0.25 μm; Agilent Technologies). The injection volume was set to 1 μL. Helium was used as a carrier gas and its flow rate was 1.14 mL/min. Split mode was applied with an inlet temperature of 250 °C and 50:1 split ratio. The GC column temperature was controlled as follows: 60 °C for 3.5 min, ramping up at 10 °C/min to 325 °C, and then holding for 10 min. MS was operated in electron impact mode with temperatures of 250 °C for the interface and 200 °C for the ion source. Analysed *m*/*z* values of the target metabolite fragment ions are summarised in Supplemental Table S2. MIDs of each target metabolite were calculated based on height values and corrected for natural isotope abundance.

### ^13^C metabolic flux analysis

A developed metabolic network model was composed of central carbon metabolic pathways (glycolysis, TCA cycle, oxidative pentose phosphate pathway, anaplerotic pathways and amino acid metabolism) and a biomass-producing reaction^33–37^. Dry cell weight was set to 514 pg/cell based on a previous report^35^, in which the metabolite coefficients were determined for the lumped biomass production reaction. Pyruvate, citrate, oxaloacetate and acetyl-CoA were separated into mitochondrial and cytosolic compartments. Regarding mitochondrial pyruvate, we included two pools in the model to represent possible connections of mitochondrial pyruvate to the TCA cycle and anaplerotic pathways^32,38^. Moreover, we introduced a mixing pool for the compartmentalized metabolites to represent relative contribution of each compartment without affecting the flux distribution in the whole network model^39^. A detailed metabolic network model is presented in Supplemental Table S1. ^13^C-MFA of parallel labelling experiments was performed using a Python version of OpenMebius^40^ implemented in Python 2.7.8 with NumPy1.9.1, SciPy 0.15.1, PyOpt 1.2 and parallel Python 1.6.4 modules. Metabolic flux values were determined by minimising the residual sum of squares (RSS) between experimentally measured and simulated MIDs using the SLSQP (sequential least squares programming) function implemented in PyOpt1.2^41^. The metabolite fragment ions used for the fitting analysis are summarised in Supplemental Table S3. The standard deviations of MID measurements were set to 0.01. Glucose uptake and lactate secretion flux were fitted to each observed value with 5% relative error and the other measured extracellular fluxes were fixed to each observed value. To assess the goodness of fit for the obtained best fitted result, a chi-squared test was applied using α of 0.05. The 95% confidence intervals for each estimated flux were estimated by the grid search method^42^. The P/O ratio for calculating ATP production flux from OxPHOS was set to 2.3^43^.

### Western blotting

After cells had been washed with DPBS, 0.2 mL of the lysis buffer (150 mM NaCl, 1% Triton-X, 50 mM Tris-HCl) containing protease inhibitor (Sigma-Aldrich Co., LLC) was added to the 60 mm dish. The cell lysate was retrieved and incubated at 4 °C for 30 min, followed by centrifugation at 15,000 rpm and 4 °C for 5 min to remove the cell debris. The resulting supernatant was incubated with NuPage LDS sample buffer (Thermo Fisher Scientific, Inc.) and NuPage Sample Reducing Agent (Thermo Fisher Scientific, Inc.) at 100 °C for 10 min. The western blot sample was applied to an acrylamide gel (D.R.C. Co., Ltd.) and separated under conditions with a constant voltage of 150 V for 90 min. Then, protein was transferred to a polyvinylidene difluoride (PVDF) membrane (Millipore) in a transfer buffer consisting of 1 × Tris-Glycine Buffer (Sigma-Aldrich Co., LLC) with 20% methanol under conditions with a constant current of 200 mA for 120 min. Protein transferred to the PVDF membrane was blocked in 5% skim milk for 60 min and treated with primary antibody at 4 °C overnight. After being washed with TBS Tween-20 five times, the membrane was treated with secondary antibody conjugated with horseradish peroxidase at 4 °C for 60 min. Then, the membrane was washed five times with TBS-T. Finally, the substrate for peroxidase (Luminata Forte Western HRP substrate; Millipore) was added to the membrane and the luminal intensity was detected with LAS4000 (Fujifilm Corporation). The antibodies used were as follows: FH, rabbit monoclonal (#4567; Cell Signaling Technology Inc.); β-actin, rabbit monoclonal (#4970; Cell Signaling Technology Inc.); GAPDH, rabbit monoclonal (#2118; Cell Signaling Technology Inc.); COX IV, rabbit monoclonal (#4850; Cell Signaling Technology Inc.); anti-rabbit IgG antibody conjugated with horseradish peroxidase (#NA934V; GE Healthcare); and anti-rabbit IgG, horseradish peroxidase-linked antibody (#7074; Cell Signaling Technology Inc.).

### FH activity assay

Cells were cultured in DMEM without glucose, glutamine, phenol red, sodium pyruvate and sodium bicarbonate (Sigma-Aldrich Co., LLC) supplemented with 20 mM glucose, 2 mM glutamine, 44 mM sodium bicarbonate and 10% FBS (HyClone Laboratories, Inc.). Mitochondrial and cytosolic fractionation was performed using a commercially available kit (Cell Fractionation Kit-Standard; Abcam). Briefly, 3.0 × 10^6^ cells were suspended in the attached Buffer A and treated with Detergent I. After incubation at room temperature for 7 min, the sample was centrifuged at 5,000×*g* and 4 °C for 1 min, after which the supernatant containing the cytosolic fraction was collected. The resulting cell pellet was resuspended in Buffer A and treated with Detergent II. After incubation at room temperature for 10 min, the sample was centrifuged at 5,000×*g* and 4 °C for 1 min, after which the supernatant containing the mitochondrial fraction was collected. Each mitochondrial and cytosolic fraction sample was aliquoted for western blotting and FH activity assay using Colorimetric Fumarase Activity Assay Kit (Abcam). For FH activity assay, after the sample had been mixed with the attached substrate, enzyme mix and developer solution, absorbance at 450 nm was measured using Versamax (Molecular Devices, LLC) in the kinetic mode at 37 °C for 120 min.

### ATP assay in glucose- or glutamine-free conditions

Parental and FH^dim^ cells were seeded into a 96-well plate at 3.5 × 10^3^ cells/well and 5.0 × 10^3^ cells/well, respectively. Culture medium was replaced with the complete medium, no glucose medium or no glutamine medium 1 day after the cell seeding (defined as day 0). From day 0 to day 3, cellular ATP was quantified as a luminescent signal using the CellTiter-Glo 2.0 Assay (Promega Corp.) and EnVision (PerkinElmer, Co., Ltd.).

### Cell viability assay

Parental and FH^dim^ cells were seeded into a 96-well plate at 2.0 × 10^3^ cells/well and 3.0 × 10^3^ cells/well, respectively. The cells were treated with oligomycin (Sigma-Aldrich Co., LLC) at concentrations of 10, 5, 2.5, 1.3, 0.63, 0.31, 0.16, 0.078 and 0.039 nM 1 day after the cell seeding (defined as day 0). Control cells were treated with 0.1% dimethyl sulfoxide. On day 3, cellular ATP was quantified as a luminescent signal using the CellTiter-Glo 2.0 Assay (Promega Corp.) and EnVision (PerkinElmer, Co., Ltd.) to determine the cell viability. Cell viability at each concentration of oligomycin was calculated using the measured luminescent signal as a percentage of the control.

## Supplementary information

Supplementary file1.

## References

[1] Tomlinson IP (2002). Germline mutations in FH predispose to dominantly inherited uterine fibroids, skin leiomyomata and papillary renal cell cancer. Nat. Genet..

[2] Sudarshan S, Pinto PA, Neckers L, Linehan WM (2007). Mechanisms of disease: hereditary leiomyomatosis and renal cell cancer—a distinct form of hereditary kidney cancer. Nat. Clin. Pract. Urol..

[3] Linehan W (2004). Genetic basis of cancer of the kidney: disease-specific approaches to therapy. Clin. Cancer Res..

[4] Yang Y (2010). UOK 262 cell line, fumarate hydratase deficient (FH-/FH-) hereditary leiomyomatosis renal cell carcinoma: in vitro and in vivo model of an aberrant energy metabolic pathway in human cancer. Cancer Genet. Cytogenet..

[5] Yang Y (2013). Metabolic reprogramming for producing energy and reducing power in fumarate hydratase null cells from hereditary leiomyomatosis renal cell carcinoma. PLoS ONE.

[6] Frezza C (2011). Haem oxygenase is synthetically lethal with the tumour suppressor fumarate hydratase. Nature.

[7] O’Flaherty L (2010). Dysregulation of hypoxia pathways in fumarate hydratase-deficient cells is independent of defective mitochondrial metabolism. Hum. Mol. Genet..

[8] Adam J (2013). A role for cytosolic fumarate hydratase in urea cycle metabolism and renal neoplasia. Cell Rep..

[9] Zheng L (2013). Reversed argininosuccinate lyase activity in fumarate hydratase-deficient cancer cells. Cancer Metab..

[10] Gonçalves E (2018). Post-translational regulation of metabolism in fumarate hydratase deficient cancer cells. Metab. Eng..

[11] Antoniewicz MR (2018). A guide to ^13^C metabolic flux analysis for the cancer biologist. Exp. Mol. Med..

[12] Wiechert W (2001). ^13^C metabolic flux analysis. Metab. Eng..

[13] Yogev O, Naamati A, Pines O (2011). Fumarase: a paradigm of dual targeting and dual localized functions. FEBS J..

[14] Dik E, Naamati A, Asraf H, Lehming N, Pines O (2016). Human fumarate hydratase is dual localized by an alternative transcription initiation mechanism. Traffic.

[15] Henry O, Jolicoeur M, Kamen A (2011). Unraveling the metabolism of HEK-293 cells using lactate isotopomer analysis. Bioprocess. Biosyst. Eng..

[16] Isaacs JS (2005). HIF overexpression correlates with biallelic loss of fumarate hydratase in renal cancer: novel role of fumarate in regulation of HIF stability. Cancer Cell.

[17] Koivunen P (2007). Inhibition of hypoxia-inducible factor (HIF) hydroxylases by citric acid cycle intermediates: possible links between cell metabolism and stabilization of HIF. J. Biol. Chem..

[18] Schmidt C, Sciacovelli M, Frezza C (2020). Fumarate hydratase in cancer: A multifaceted tumour suppressor. Semin. Cell Dev. Biol..

[19] Yang M, Soga T, Pollard PJ, Adam J (2012). The emerging role of fumarate as an oncometabolite. Front. Oncol..

[20] Zheng L (2015). Fumarate induces redox-dependent senescence by modifying glutathione metabolism. Nat. Commun..

[21] Phang JM, Liu W, Hancock CN, Fischer JW (2015). Proline metabolism and cancer: emerging links to glutamine and collagen. Curr. Opin. Clin. Nutr. Metab. Care.

[22] De Ingeniis J (2012). Functional specialization in proline biosynthesis of melanoma. PLoS ONE.

[23] Grassian AR (2014). IDH1 mutations alter citric acid cycle metabolism and increase dependence on oxidative mitochondrial metabolism. Cancer Res..

[24] Hollinshead KER (2018). Oncogenic IDH1 mutations promote enhanced proline synthesis through PYCR1 to support the maintenance of mitochondrial redox homeostasis. Cell Rep..

[25] Sudarshan S (2009). Fumarate hydratase deficiency in renal cancer induces glycolytic addiction and hypoxia-inducible transcription factor 1alpha stabilization by glucose-dependent generation of reactive oxygen species. Mol. Cell. Biol..

[26] Tyrakis PA (2017). Fumarate hydratase loss causes combined respiratory chain defects. Cell Rep..

[27] Muir A, Danai LV, Vander Heiden MG (2018). Microenvironmental regulation of cancer cell metabolism: implications for experimental design and translational studies. Dis. Model. Mech..

[28] Nunes AS, Barros AS, Costa EC, Moreira AF, Correia IJ (2019). 3D tumor spheroids as in vitro models to mimic in vivo human solid tumors resistance to therapeutic drugs. Biotechnol. Bioeng..

[29] Armenta JM (2010). Sensitive and rapid method for amino acid quantitation in malaria biological samples using AccQ.Tag ultra performance liquid chromatography-electrospray ionization-MS/MS with multiple reaction monitoring. Anal. Chem..

[30] Glacken MW, Adema E, Sinskey AJ (1988). Mathematical descriptions of hybridoma culture kinetics: I Initial metabolic rates. Biotechnol. Bioeng..

[31] Ozturk SS, Palsson BO (1990). Chemical decomposition of glutamine in cell culture media: effect of media type, pH, and serum concentration. Biotechnol. Prog..

[32] Ahn WS, Antoniewicz MR (2013). Parallel labeling experiments with [1,2–^13^C]glucose and [U-^13^C]glutamine provide new insights into CHO cell metabolism. Metab. Eng..

[33] Okahashi N (2015). Metabolic characterization of cultured mammalian cells by mass balance analysis, tracer labeling experiments and computer-aided simulations. J. Biosci. Bioeng..

[34] Araki C, Okahashi N, Maeda K, Shimizu H, Matsuda F (2018). Mass spectrometry-based method to study inhibitor-induced metabolic redirection in the central metabolism of cancer cells. Mass Spectrom. (Tokyo).

[35] Dietmair S (2012). A multi-omics analysis of recombinant protein production in HEK293 cells. PLoS ONE.

[36] Sheikh K, Förster J, Nielsen LK (2005). Modeling hybridoma cell metabolism using a generic genome-scale metabolic model of *Mus musculus*. Biotechnol. Prog..

[37] Keibler MA (2016). Metabolic requirements for cancer cell proliferation. Cancer Metab..

[38] Lu D (2002). ^13^C NMR isotopomer analysis reveals a connection between pyruvate cycling and glucose-stimulated insulin secretion (GSIS). Proc. Natl. Acad. Sci. USA.

[39] Noguchi Y, Young JD, Aleman JO, Hansen ME, Kelleher JK, Stephanopoulos G (2009). Effect of anaplerotic fluxes and amino acid availability on hepatic lipoapoptosis. J. Biol. Chem..

[40] Kajihata, S., Furusawa, C., Matsuda, F., Shimizu, H. OpenMebius: an open source software for isotopically nonstationary ^13^C-based metabolic flux analysis. *Biomed. Res. Int.* 627014 (2014). 10.1155/2014/627014 PMC4071984 25006579

[41] Ruben EP, Peter WJ, Joaquim RRAM (2012). pyOpt: a Python-based object-oriented framework for nonlinear constrained optimization. Struct. Multidiscip. Optim..

[42] Maeda K, Okahashi N, Toya Y, Matsuda F, Shimizu H (2016). Investigation of useful carbon tracers for ^13^C-metabolic flux analysis of Escherichia coli by considering five experimentally determined flux distributions. Metab. Eng. Commun..

[43] Hinkle PC (2005). P/O ratios of mitochondrial oxidative phosphorylation. Biochim. Biophys. Acta.

