# Supplementary material for: Direct and quantitative analysis of altered metabolic flux distributions and cellular ATP production pathway in fumarate hydratase-diminished cells
Source: Sci Rep. 2020 Aug 3;10:13065. doi: 10.1038/s41598-020-70000-6 (PMC7400513; doi:10.1038/s41598-020-70000-6)
Supplement: Supplementary file 1 — Supplementary file1. [file 41598_2020_70000_MOESM1_ESM.pdf]

Direct and quantitative analysis of altered metabolic flux distributions and cellular ATP production pathway in fumarate hydratase-diminished cells

Shingo Noguchi[1,2], Hirokazu Ishikawa[3], Kenichi Wakita[1], Fumio Matsuda[2] and Hiroshi Shimizu[2,*]

[1]Biomarker Department, Daiichi Sankyo Co., Ltd., Tokyo, Japan

[2]Department of Bioinformatic Engineering, Graduate School of Information Science and Technology, Osaka University, Osaka, Japan

[3]Biological Research Department, Daiichi Sankyo RD Novare Co., Ltd., Tokyo, Japan

[*]Corresponding author: shimizu@ist.osaka-u.ac.jp

Supplemental Figure S1. Time-dependent change of MID in each metabolite labelled by [1,2-^{13}C]glucose. Each data point and error bar represent the mean and standard deviation from triplicate samples.

Supplemental Figure S2. Time-dependent change of MID in each metabolite labelled by [U-^{13}C]glutamine. Each data point and error bar represent the mean and standard deviation from triplicate samples.

Supplemental Figure S3. Full length images of western blotting. Red frame represents cropped regions in Figure 1c and Figure 1d in the main manuscript.

Supplemental Table S1. Determined metabolic flux distributions in parental and FH^{dim} cells. LB, lower boundary; UB, upper boundary.

Supplemental Table S2. List of ions used for GC-MS measurement.

Supplemental Table S3. Measured and simulated MIDs for fitting analysis.

Supplemental Table S4. NADPH production and consumption flux.

Supplemental Table S5. NADH production and consumption flux.

Supplemental Table S6. ATP production and consumption flux.

Supplemental Figure S1

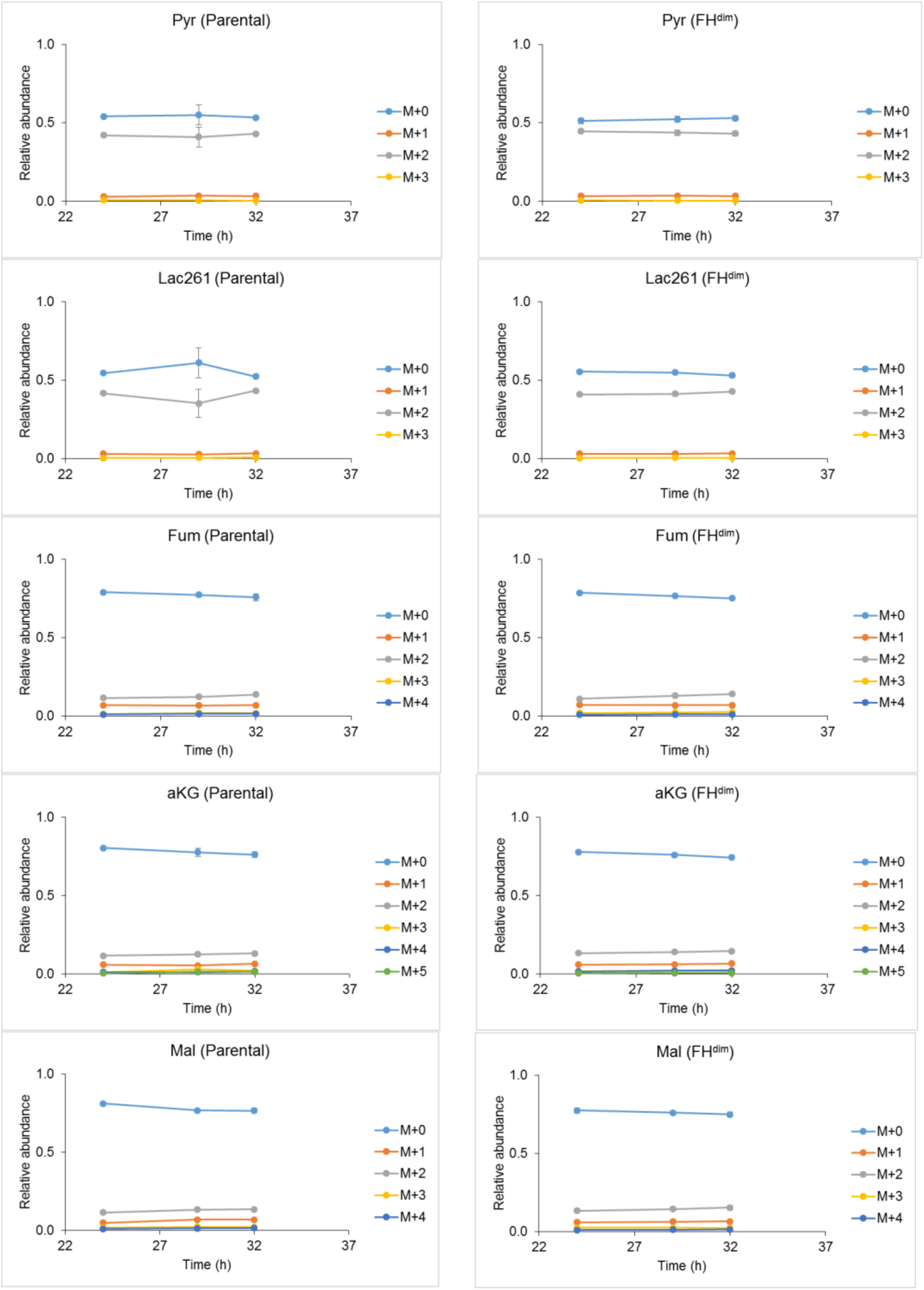

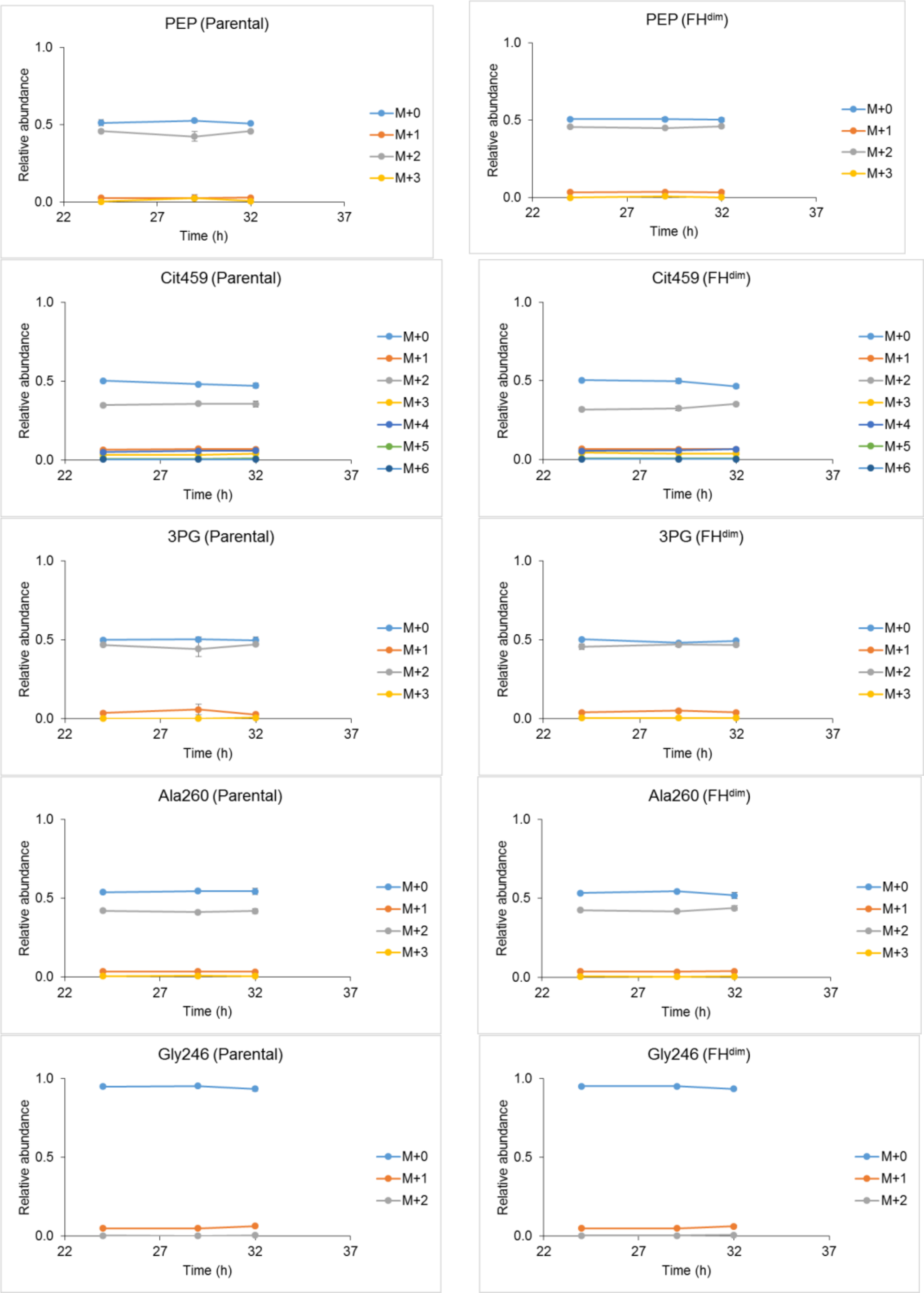
PEP (Parental)
PEP (FHdim)
Cit459 (Parental)
Cit459 (FHdim)
3PG (Parental)
3PG (FHdim)
Ala260 (Parental)
Ala260 (FHdim)
Gly246 (Parental)
Gly246 (FHdim)
Relative abundance
Time (h)
1.0
0.5
0.0
22
27
32
37
M+0
M+1
M+2
M+3
M+4
M+5
M+6

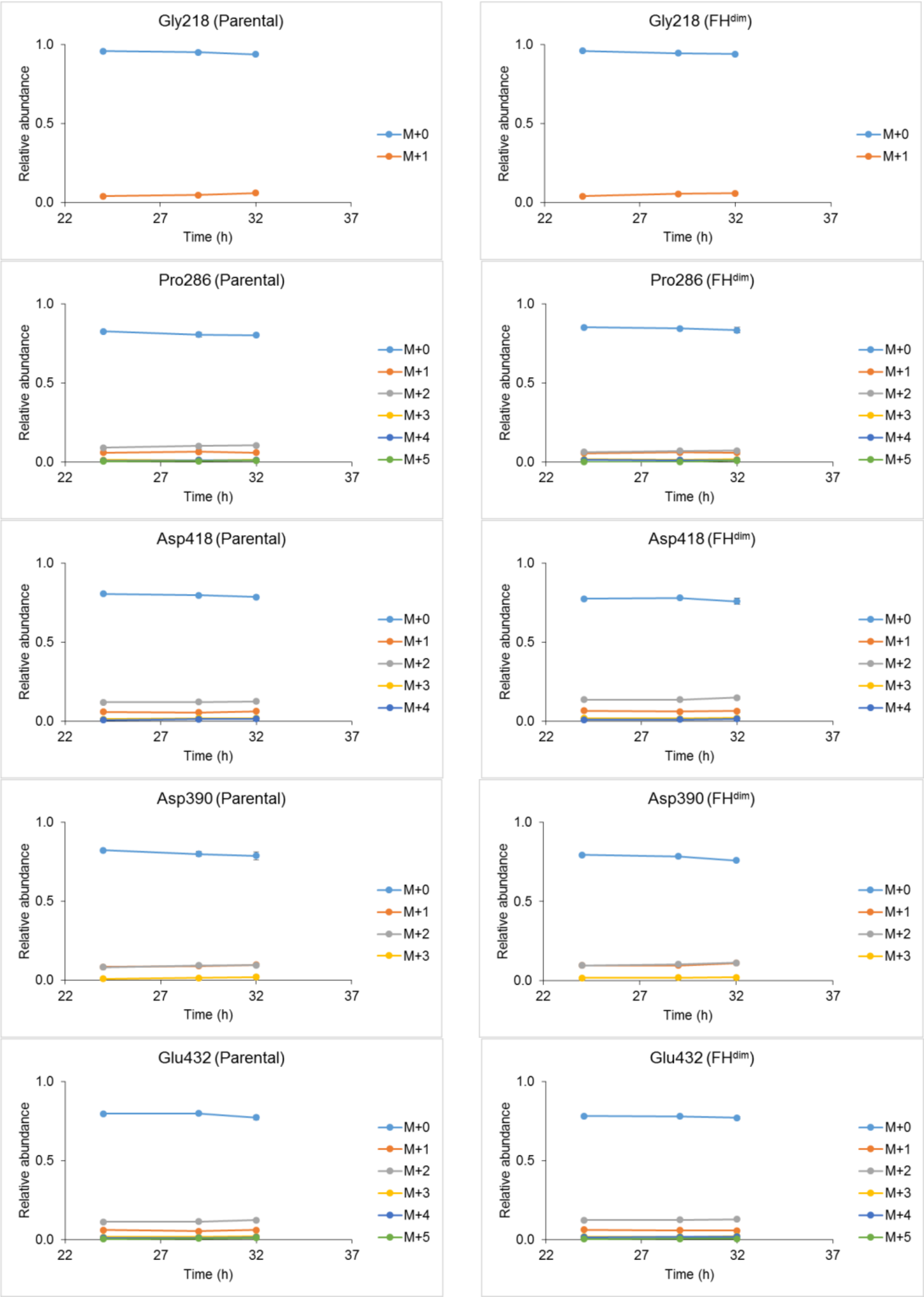
Gly218 (Parental)
Gly218 (FHdim)
Pro286 (Parental)
Pro286 (FHdim)
Asp418 (Parental)
Asp418 (FHdim)
Asp390 (Parental)
Asp390 (FHdim)
Glu432 (Parental)
Glu432 (FHdim)
Relative abundance
1.0
0.5
0.0
22
27
32
37
Time (h)
M+0
M+1
M+2
M+3
M+4
M+5

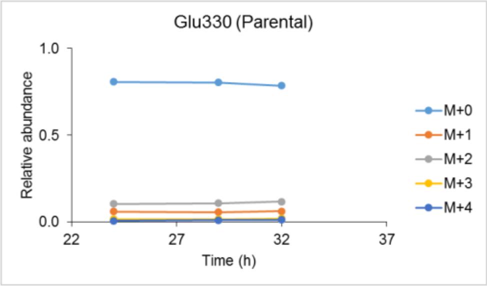

Glu330 (Parental)
Relative abundance
1.0
0.5
0.0
22
27
32
37
Time (h)
M+0
M+1
M+2
M+3
M+4

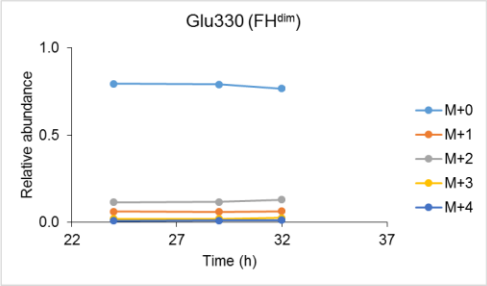

Glu330 (FH^{dim})
Relative abundance
1.0
0.5
0.0
22
27
32
37
Time (h)
M+0
M+1
M+2
M+3
M+4

Supplemental Figure S2

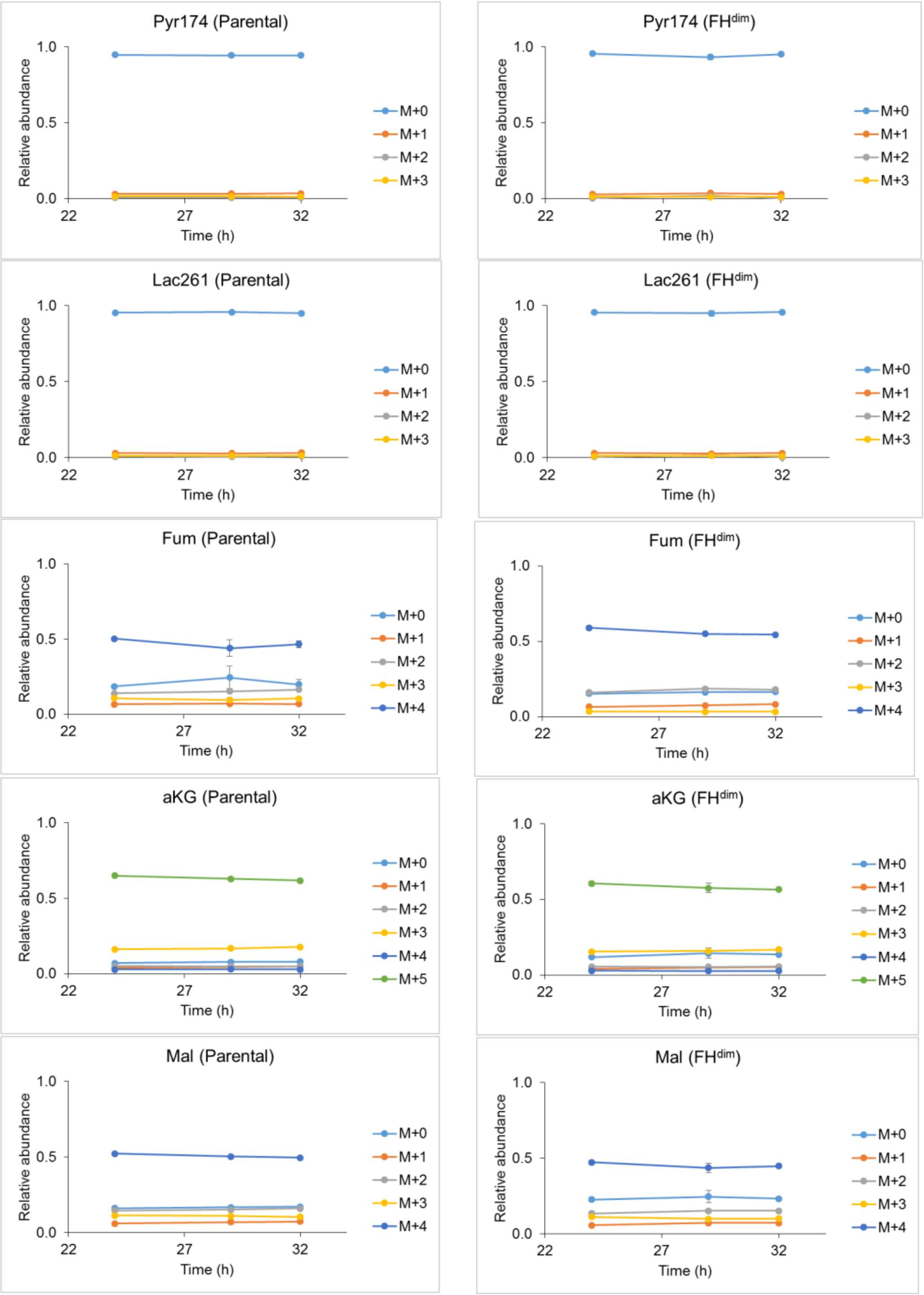

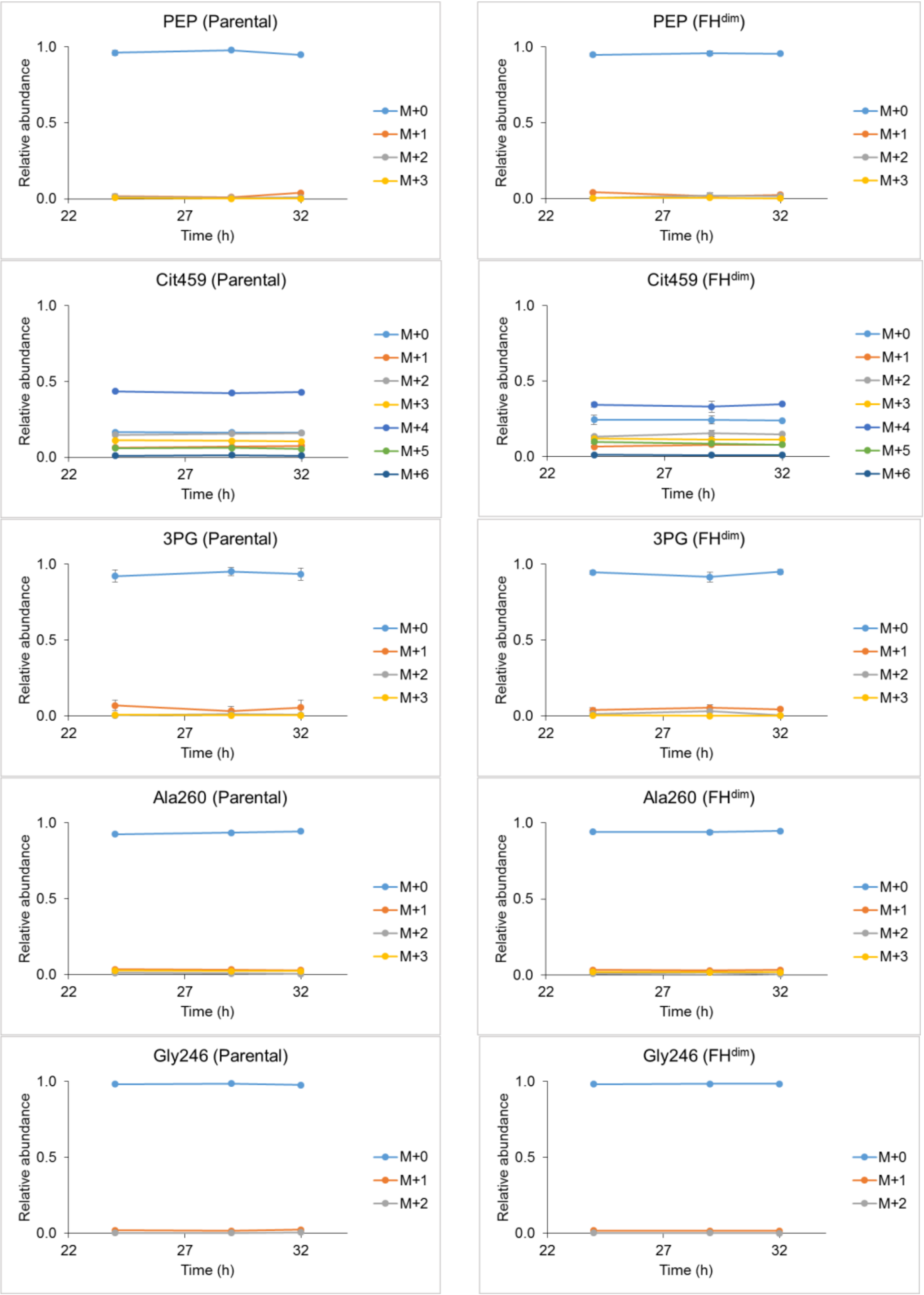

PEP (Parental)
PEP (FHdim)
Cit459 (Parental)
Cit459 (FHdim)
3PG (Parental)
3PG (FHdim)
Ala260 (Parental)
Ala260 (FHdim)
Gly246 (Parental)
Gly246 (FHdim)
Relative abundance
Time (h)
1.0
0.5
0.0
22
27
32
M+0
M+1
M+2
M+3
M+4
M+5
M+6

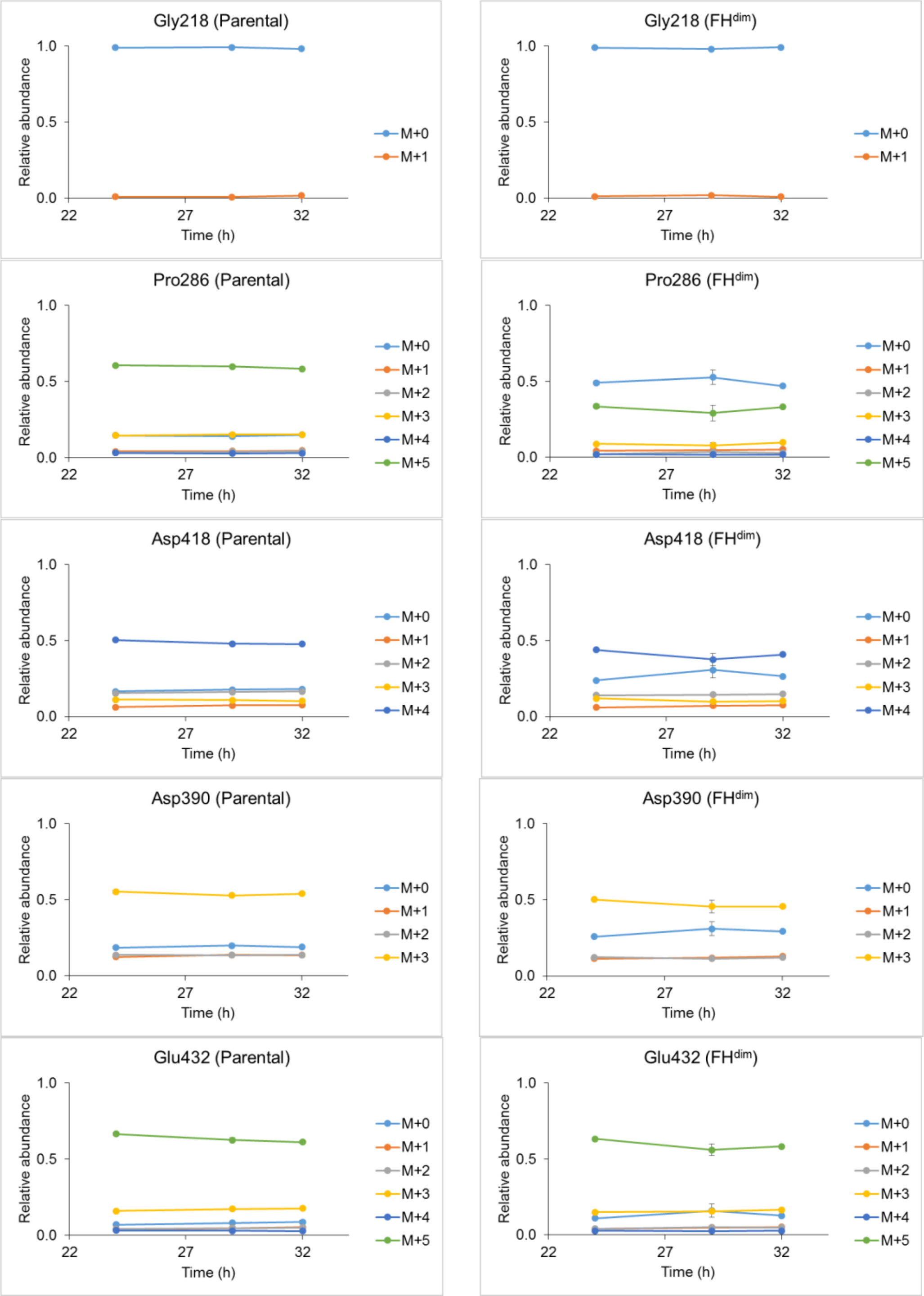
Gly218 (Parental)
Gly218 (FHdim)
Pro286 (Parental)
Pro286 (FHdim)
Asp418 (Parental)
Asp418 (FHdim)
Asp390 (Parental)
Asp390 (FHdim)
Glu432 (Parental)
Glu432 (FHdim)
Relative abundance
1.0
0.5
0.0
22
27
32
Time (h)
M+0
M+1
M+2
M+3
M+4
M+5

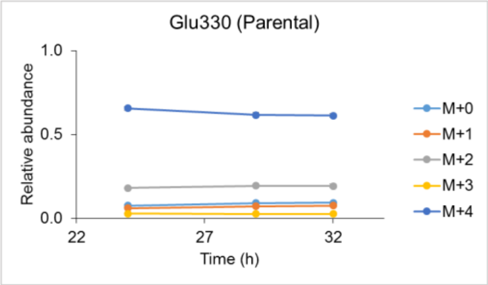

Glu330 (Parental)
1.0
0.5
0.0
Relative abundance
22
27
32
Time (h)
M+0
M+1
M+2
M+3
M+4

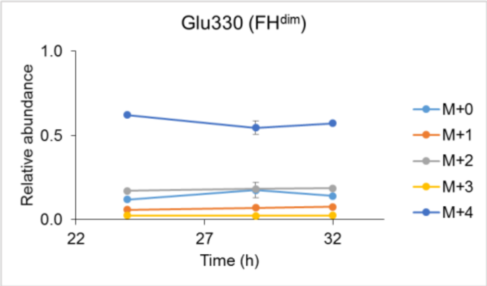

Glu330 (FHdim)
1.0
0.5
0.0
Relative abundance
22
27
32
Time (h)
M+0
M+1
M+2
M+3
M+4

Supplemental Figure S3

Figure 1c

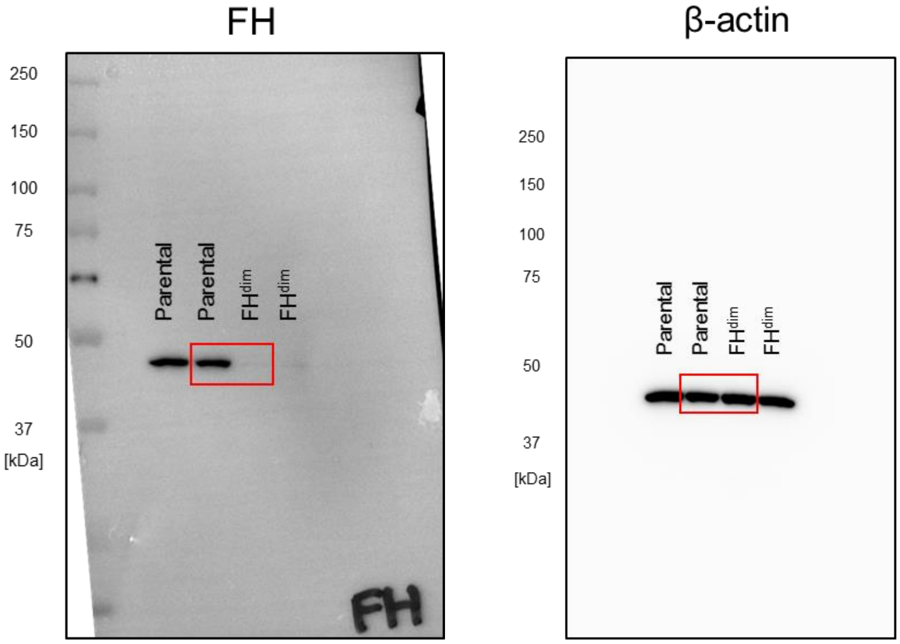

Figure 1d

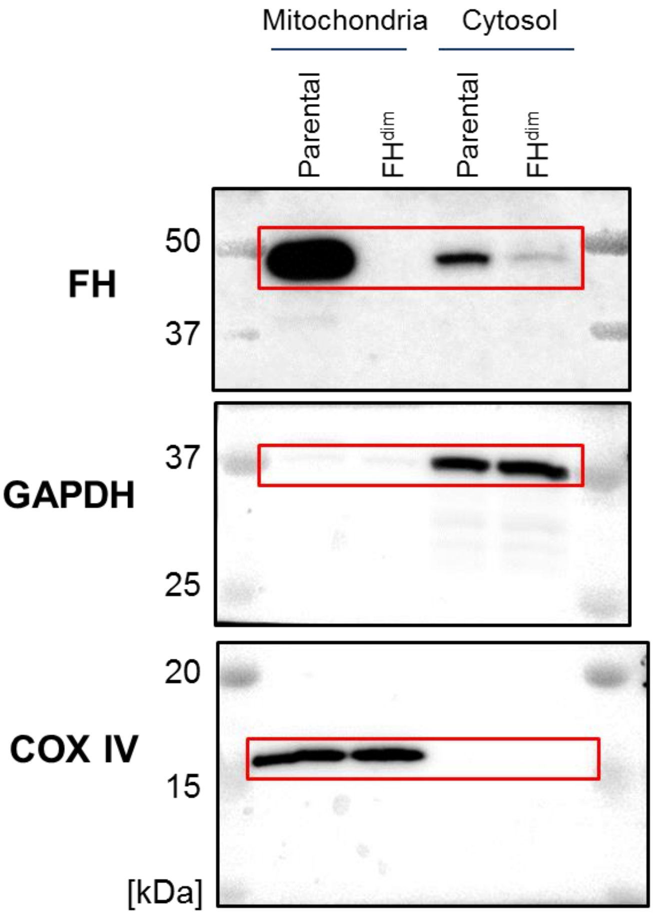

Supplemental Table S1

Reaction ID	Reaction	Parental [nmol/10^6 cells/h]			FH^{dim} [nmol/10^6 cells/h]		
		Flux	LB	UB	Flux	LB	UB
SubsGlc	SubsGlc-->G6P	607.5	539.7	686.4	558.6	504.1	633.6
SubsArg	SubsArg-->Arg	8.7	-	-	9.6	-	-
SubsCys	SubsCys-->Cys	4.1	-	-	3.3	-	-
SubsGln	SubsGln-->Gln	82.7	-	-	52.7	-	-
SubsSer	SubsSer-->Ser	22.0	-	-	16.2	-	-
SubsCO2	SubsCO2-->CO2in	8869.3	-	-	369186.6	-	-
Lac_ex	Lac-->LacEx	1044.5	866.4	1206.7	983.7	863.4	1133.2
Pyr_ex	Pyrcyt-->PyrEx	61.7	-	-	57.8	-	-
Ala_ex	Ala-->AlaEx	17.1	-	-	19.4	-	-
Pro_ex	Pro-->ProEx	13.9	-	-	1.5	-	-
Cit_Dummy	Cit-->Citdummy	100.0	-	-	100.0	-	-
Pyr_Dummy	Pyr-->Pyrdummy	100.0	-	-	100.0	-	-
aKG_Dummy	aKG-->aKGdummy	100.0	-	-	100.0	-	-
Glycolysis_r1	G6P-->F6P	539.6	-	-	488.7	-	-
Glycolysis_r1r	F6P-->G6P	0.0	-	-	0.0	-	-
Glycolysis_r2	F6P-->FBP	578.9	527.6	633.6	531.6	490.5	581.9
Glycolysis_r3	FBP-->DHAP+GAP	685.3	-	-	8053.1	-	-
Glycolysis_r3r	DHAP+GAP-->FBP	106.4	-	-	7521.5	-	-
Glycolysis_r4	DHAP-->GAP	49402.3	-	-	207737.2	-	-
Glycolysis_r4r	GAP-->DHAP	48825.6	-	-	207206.8	-	-
Glycolysis_r5	GAP-->PGA	1175.3	-	-	2953.6	-	-
Glycolysis_r5r	PGA-->GAP	0.0	-	-	1870.1	-	-
Glycolysis_r6	PGA-->PEP	1205.5	-	-	2969.4	-	-
Glycolysis_r6r	PEP-->PGA	33.0	-	-	1888.1	-	-
Glycolysis_r7	PEP-->Pyrcyt	1172.4	1071.5	1281.9	1081.4	999.6	1182.5
Glycolysis_r8	Pyrcyt-->Lac	1044.5	942.8	1153.8	983.7	901.8	1084.9
TCA_r1	Pyrcyt-->Pyrmit	108.6	88.3	117.1	57.8	54.7	75.8
TCA_r2	Pyrmit-->AcCOAmit+CO2in	80.1	74.5	87.5	53.1	49.9	56.3
TCA_r3	AcCOAmit+Oxamit-->Citmit	80.1	74.2	87.3	53.1	49.8	56.3
TCA_r4	Citmit-->aKGmit+CO2in	46.8	-	-	34.4	-	-

TCA_r4r	aKGmit+CO2in-->Citmit	0.0	-	-	0.0	-	-
TCA_r5	aKGmit-->Suc+CO2in	89.6	83.7	97.0	72.7	69.4	75.9
TCA_r6	Suc-->Fum	23194.0	-	-	412.9	-	-
TCA_r6r	Fum-->Suc	23104.4	-	-	340.2	-	-
TCA_r7	Fum-->Mal	194650.0	-	-	89.6	-	-
TCA_r7r	Mal-->Fum	194560.3	-	-	16.9	-	-
TCA_r8	Mal-->Oxamit	446715.0	-	-	180.7	-	-
TCA_r8r	Oxamit-->Mal	446640.5	-	-	137.1	-	-
Anaplerosis_r1	Pyrmit2+CO2in-->Oxamit	17.0	12.4	27.4	16.3	13.1	20.2
Anaplerosis_r2	Mal-->Pyrmit2+CO2in	13.8	4.5	27.3	11.7	7.3	16.7
Anaplerosis_r3	Mal-->Pyrcyt+CO2in	44.7	38.8	50.4	43.6	39.7	47.3
Anaplerosis_r4	Pyrmit-->Pyrmit2	59.0	-	-	33.2	-	-
Anaplerosis_r4r	Pyrmit2-->Pyrmit	55.8	-	-	28.6	-	-
PPP_r1	G6P-->Ru5P+CO2in	62.9	20.4	92.4	66.9	24.7	106.3
PPP_r2	Ru5P-->R5P	24.6	-	-	5431.5	-	-
PPP_r2r	R5P-->Ru5P	0.8	-	-	5407.5	-	-
PPP_r3	Ru5P-->Xu5P	9207.6	-	-	26407.5	-	-
PPP_r3r	Xu5P-->Ru5P	9168.4	-	-	26364.5	-	-
PPP_r4	R5P+Xu5P-->S7P+GAP	19.6	-	-	21.5	-	-
PPP_r4r	GAP+S7P-->Xu5P+R5P	0.0	-	-	0.0	-	-
PPP_r5	GAP+S7P-->F6P+E4P	273.3	-	-	14761.8	-	-
PPP_r5r	E4P+F6P-->S7P+GAP	253.6	-	-	14740.3	-	-
PPP_r6	E4P+Xu5P-->F6P+GAP	53.6	-	-	49.1	-	-
PPP_r6r	GAP+F6P-->Xu5P+E4P	34.0	-	-	27.7	-	-
AA_r1	Gln-->Glu	77.0	-	-	49.2	-	-
AA_r2	Glu-->aKGmit	9035.8	-	-	1998.0	-	-
AA_r2r	aKGmit-->Glu	8982.9	-	-	1952.1	-	-
AA_r3	Glu-->Pro	17.9	17.5	18.3	2.8	2.7	2.9
AA_r4	Oxamit-->Asp	11.4	-	-	6.9	-	-
AA_r5	Asp-->Asn	5.1	-	-	3.1	-	-
AA_r6	Ser-->Gly	18.9	-	-	15.6	-	-
AA_r7	Gly-->GlyDeg	9.4	-	-	9.8	-	-
AA_r8	PGA-->Ser	2.8	-	-	2.1	-	-
AA_r8r	Ser-->PGA	0.0	-	-	0.0	-	-
AA_r9	Arg-->Glu	0.6	0.2	1.0	3.6	3.5	3.7

AA_r10	Cys-->Ser	1.6	-	-	1.8	-	-
AA_r11	Arg-->Pro	1.5	1.1	1.9	2.0	1.9	2.1
AA_r12	Pyrmit-->Ala	25.3	-	-	0.0	-	-
AA_r13	Pyrcyt-->Ala	2.4	-	-	25.8	-	-
Cyt_r1	Citcyt-->AcCOAcyt+Oxacyt	43.4	-	-	26.3	-	-
Cyt_r2	Mal-->Oxacyt	18112.5	-	-	499.1	-	-
Cyt_r2r	Oxacyt-->Mal	18155.9	-	-	525.4	-	-
Cyt_r3	Citmit-->Citcyt	33.3	-	-	18.7	-	-
Cyt_r4	aKGmit-->aKGcyt	10.1	1.4	14.7	7.7	2.4	10.3
Cyt_r5	aKGcyt+CO2in-->Citcyt	10.1	1.4	14.7	7.7	2.3	10.3
BIOMASS	{308.4}Ala+{193.8}Arg+{184.5}Asp+{148}Asn+{74.5}Cys+{165.5}Gln+{198.4}Glu+{276.5}Gly+{160.9}Pro+{221.0}Ser+{1265.6}AcCOAcyt+{143.1}G6P+{119.4}R5P+{61.2}DHAP-->Biomass	0.034	-	-	0.021	-	-
CO2	CO2in-->CO2Ex	9180.1	-	-	369445.1	-	-
Mix_r1	{0}Citmit-->Cit	83.0	-	-	69.9	-	-
Mix_r2	{0}Citcyt-->Cit	17.0	-	-	30.1	-	-
Mix_r3	{0}Pyrmit-->Pyr	0.0	-	-	0.0	-	-
Mix_r4	{0}Pyrmit2-->Pyr	0.0	-	-	0.0	-	-
Mix_r5	{0}Pyrcyt-->Pyr	100.0	-	-	100.0	-	-
Mix_r6	{0}aKGmit-->aKG	41.4	-	-	49.4	-	-
Mix_r7	{0}aKGcyt-->aKG	58.6	-	-	50.6	-	-
PEPH	Glycolysis_r6<=>Glycolysis_r6r	1172.4	1073.2	1281.8	1081.4	999.6	1182.5
MDH	TCA_r8<=>TCA_r8r	74.5	67.6	80.4	43.7	38.9	48.1
TKT2	PPP_r6<=>PPP_r6r	19.6	5.4	29.4	21.5	7.4	34.6
TKT1	PPP_r4<=>PPP_r4r	19.6	5.5	29.4	21.5	7.4	34.6
PyrmitExchange	Anaplerosis_r4<=>Anaplerosis_r4r	3.2	-2.8	8.9	4.6	0.7	8.2
GAPDH	Glycolysis_r5<=>Glycolysi	1175.3	1075.4	1281.4	1083.5	1001.9	1184.6

	s_r5r						
TPI	Glycolysis_r4<=>Glycolysis_r4r	576.8	526.3	631.8	530.4	489.2	580.6
PGI	Glycolysis_r1<=>Glycolysis_r1r	539.6	474.1	613.7	488.7	437.0	551.7
TAL	PPP_r5<=>PPP_r5r	19.6	5.4	29.4	21.5	7.4	34.6
FBA	Glycolysis_r3<=>Glycolysis_r3r	578.9	527.5	632.5	531.6	490.5	581.9
IDH	TCA_r4<=>TCA_r4r	46.8	43.2	50.5	34.4	29.5	37.2
GLUDH	AA_r2<=>AA_r2r	52.9	-	-	45.9	-	-
RPI	PPP_r2<=>PPP_r2r	23.7	9.5	33.5	24.0	9.9	37.1
MDHcyt	Cyt_r2<=>Cyt_r2r	-43.4	-	-	-26.3	-	-
RBE	PPP_r3<=>PPP_r3r	39.2	10.9	58.9	42.9	14.8	69.2
SDH	TCA_r6<=>TCA_r6r	89.6	83.7	97.0	72.7	69.4	75.9
FH	TCA_r7<=>TCA_r7r	89.6	83.7	97.0	72.7	69.4	75.9
PGAtoSer	AA_r8<=>AA_r8r	2.8	-	-	2.1	-	-

Supplemental Table S2

Metabolites	*m/z*	Chemical formula
**MTBSTFA derivatisation**		
Pyruvate	174	$C_6H_{12}O_3NSi$
Lactate	261	$C_{11}H_{25}O_3Si_2$
Alanine	260	$C_{11}H_{26}O_2NSi_2$
Glycine	246	$C_{10}H_{24}O_2Si_2$
Glycine	218	$C_9H_{24}O_1Si_2$
Lactate	261	$C_{11}H_{25}O_3Si_2$
Fumarate	287	$C_{12}H_{23}O_4Si_2$
α-Ketoglutarate	346	$C_{14}H_{28}O_5N_1Si_2$
Malate	419	$C_{18}H_{39}O_5Si_3$
Phosphoenolpyruvate	453	$C_{17}H_{38}O_6Si_3P_1$
Citrate	459	$C_{20}H_{39}O_6Si_3$
3-Phosphoglycerate	585	$C_{23}H_{54}O_7Si_4P_1$
Proline	286	$C_{13}H_{28}O_2N_1Si_2$
Proline	184	$C_{10}H_{22}N_1Si_1$
Aspartate	418	$C_{18}H_{40}O_4N_1Si_3$
Aspartate	390	$C_{17}H_{40}O_3N_1Si_3$
Glutamate	432	$C_{19}H_{42}O_4N_1Si_3$
Glutamate	330	$C_{16}H_{36}O_2N_1Si_2$
**MSTFA derivatisation**		
Fumarate	245	$C_9H_{17}O_4Si_2$

Supplemental Table S3

^{13}C carbon source	Metabolite Name	*m/z*	Mass	Parental		FH^{dim}	
				Measured MIDs	Simulated MIDs	Measured MIDs	Simulated MIDs
[1,2-^{13}C]Glucose	3PG	585	M+0	0.514	0.497	0.515	0.492
			M+1	0.025	0.026	0.027	0.038
			M+2	0.454	0.471	0.451	0.468
			M+3	0.007	0.006	0.007	0.003
	Ala	260	M+0	0.541	0.544	0.524	0.519
			M+1	0.035	0.032	0.031	0.037
			M+2	0.416	0.418	0.438	0.439
			M+3	0.008	0.006	0.007	0.006
	Gly	218	M+0	0.943	0.940	0.944	0.941
			M+1	0.057	0.060	0.056	0.059
	Gly	246	M+0	0.931	0.932	0.933	0.934
			M+1	0.068	0.063	0.066	0.061
			M+2	0.001	0.005	0.001	0.005
	Lac	261	M+0	0.522	0.525	0.524	0.532
			M+1	0.028	0.033	0.031	0.033
			M+2	0.442	0.434	0.438	0.429
			M+3	0.007	0.008	0.007	0.007
	PEP	453	M+0	0.514	0.508	0.515	0.503
			M+1	0.025	0.028	0.027	0.035
			M+2	0.454	0.458	0.451	0.461
			M+3	0.007	0.005	0.007	0.002
	Pyr	174	M+0	0.522	0.532	0.524	0.530
			M+1	0.028	0.033	0.031	0.034
			M+2	0.442	0.430	0.438	0.432
			M+3	0.007	0.004	0.007	0.004
[U-^{13}C]Glutamine	AKG	346	M+0	0.083	0.080	0.148	0.135
			M+1	0.049	0.049	0.052	0.053
			M+2	0.054	0.050	0.054	0.055
			M+3	0.182	0.177	0.166	0.168
			M+4	0.012	0.027	0.013	0.025
			M+5	0.620	0.616	0.567	0.564

Asp	390	M+0	0.198	0.189	0.284	0.293
		M+1	0.136	0.135	0.130	0.129
		M+2	0.138	0.137	0.124	0.121
		M+3	0.527	0.539	0.462	0.457
		M+0	0.182	0.180	0.268	0.266
		M+1	0.072	0.075	0.072	0.076
		M+2	0.166	0.165	0.151	0.148
		M+3	0.101	0.102	0.102	0.103
		M+4	0.479	0.477	0.407	0.407
Cit	459	M+0	0.158	0.161	0.226	0.237
		M+1	0.070	0.077	0.073	0.076
		M+2	0.159	0.160	0.147	0.145
		M+3	0.105	0.105	0.108	0.111
		M+4	0.424	0.430	0.341	0.345
		M+5	0.051	0.056	0.074	0.077
		M+6	0.033	0.012	0.030	0.008
Fum	287	M+0	0.182	0.198	0.168	0.164
		M+1	0.072	0.069	0.072	0.082
		M+2	0.166	0.164	0.184	0.178
		M+3	0.101	0.104	0.031	0.032
		M+4	0.480	0.465	0.545	0.544
Glu	330	M+0	0.088	0.093	0.152	0.141
		M+1	0.072	0.076	0.071	0.076
		M+2	0.201	0.193	0.184	0.186
		M+3	0.015	0.027	0.015	0.025
		M+4	0.624	0.612	0.577	0.572
Glu	432	M+0	0.083	0.086	0.147	0.127
		M+1	0.049	0.051	0.051	0.051
		M+2	0.053	0.049	0.053	0.048
		M+3	0.180	0.175	0.161	0.164
		M+4	0.012	0.027	0.012	0.027
		M+5	0.623	0.612	0.576	0.583
Lac	261	M+0	0.940	0.949	0.940	0.957
		M+1	0.035	0.031	0.035	0.029
		M+2	0.005	0.007	0.006	0.003

		M+3	0.019	0.012	0.018	0.011
Mal	419	M+0	0.182	0.169	0.229	0.232
		M+1	0.072	0.073	0.072	0.070
		M+2	0.166	0.159	0.159	0.151
		M+3	0.101	0.104	0.096	0.099
		M+4	0.479	0.495	0.444	0.448
Pro	184	M+0	0.154	0.158	0.485	0.484
		M+1	0.069	0.065	0.059	0.057
		M+2	0.186	0.176	0.109	0.106
		M+3	0.014	0.027	0.009	0.016
		M+4	0.577	0.574	0.339	0.337
Pro	286	M+0	0.148	0.149	0.477	0.470
		M+1	0.049	0.046	0.051	0.052
		M+2	0.049	0.042	0.031	0.030
		M+3	0.167	0.151	0.095	0.099
		M+4	0.011	0.029	0.007	0.018
		M+5	0.576	0.584	0.338	0.332
Pyr	174	M+0	0.940	0.946	0.940	0.953
		M+1	0.035	0.034	0.035	0.031
		M+2	0.005	0.007	0.006	0.005
		M+3	0.019	0.013	0.018	0.012

Supplemental Table S4

Type	Reaction name	Stoichiometry	Parental (nmol/10^6 cells/h)	FH^{dim} (nmol/10^6 cells/h)
NADPH production flux	OxPPP	G6P + NADP --> Ru5P + CO2in + NADPH	62.9	66.9
	MEcyt	Mal + NADP --> Pyrcyt + CO2in + NADPH	44.7	43.6
	One_carbon_1	Ser + 2 NADP --> Gly + 2 NADPH	37.8	31.1
	One_carbon_2	Gly + 2 NADP --> GlyDeg + 2 NADPH	18.8	19.6
	Sum		164.2	161.3
NADPH consumption flux	Lipid synthesis	AcCOAcyt + 1.85 NADPH --> Lipid_BIOMASS + 1.85 NADP	80.3	48.7
	IDHcyt	aKGcyt+CO2in + NADPH --> Citcyt + NADP	10.1	7.7
	Pro_synthesis_1	Glu + 2 NADPH --> Pro + 2 NADP	35.8	5.6
	Pro_synthesis_2	Arg + NADPH --> Pro + NADP	1.5	2.0
	Sum		127.7	64.0
**Net NADPH production flux**			**36.5**	**97.3**

Supplemental Table S5

Type	Reaction name	Pathway	Stoichiometry	Parental (nmol/10^6 cells/h)	FH^{dim} (nmol/10^6 cells/h)
NADH production flux	PDH	TCA cycle	Pyrmit + NAD --> AcCOAmit + CO2in + NADH	80.1	53.1
	aKGDH	TCA cycle	aKGmit + NAD --> Suc + CO2in + NADH	89.6	72.7
	MEmit	Others	Mal + NAD --> Pyrmit2 + CO2in + NADH	13.8	11.7
	MDHmit	TCA cycle	Mal + NAD --> Oxamit + NADH	74.5	43.7
	GAPDH	Glycolysis	GAP + NAD --> PGA + NADH	1175.3	1083.5
	IDHmit	TCA cycle	Citmit + NAD --> aKGmit + CO2in + NADH	46.8	34.4
	SDH	TCA cycle	Suc + 0.6 NAD --> Fum + 0.6 NADH	53.8	43.6
	Sum			1533.9	1342.7
NADH consumption flux	LDH	Glycolysis	Pyrcyt + NADH --> Lac + NAD	1044.5	983.7
	MDHcyt	Others	Oxacyt + NADH --> Mal + NAD	43.4	26.3
	Pro_synthesis_1	Others	Glu + NADH --> Pro + NAD	17.9	2.8
	Pro_synthesis_2	Others	Arg + NADH --> Glu + NAD	1.5	2.0
	Sum			1107.3	1014.8
**Net NADH production flux**				**426.6**	**327.9**

Supplemental Table S6

Type	Reaction name	Pathway	Stoichiometry	Parental (nmol/10^6 cells/h)	FH^{dim} (nmol/10^6 cells/h)
ATP production flux	GAPDH	Glycolysis	GAP + ADP --> PGA + ATP	1175.3	1083.5
	aKGDH	TCA cycle	aKGmit + ADP --> Suc + CO2in + ATP	89.6	72.7
	PK	Glycolysis	PEP + ADP --> Pyrcyt + ATP	1172.4	1081.4
	OXPHOS	TCA cycle	NADH --> 2.3 ATP	981.3	754.1
	Sum			3418.6	2991.7
ATP consumption flux	HK	Glycolysis	SubsGlc + ATP --> G6P + ADP	607.5	558.6
	PFK	Glycolysis	F6P + ATP --> FBP + ADP	578.9	531.6
	PC	Others	Pyrmit2 + CO2in -+ ATP --> Oxamit + ADP	17.0	16.3
	Sum			1203.3	1106.5
**Net ATP production flux**				**2215.3**	**1885.1**
